# Novel Antifungal Activity of Q-Griffithsin, a Broad-Spectrum Antiviral Lectin

**DOI:** 10.1128/Spectrum.00957-21

**Published:** 2021-09-08

**Authors:** Henry W. Nabeta, Joseph C. Kouokam, Amanda B. Lasnik, Joshua L. Fuqua, Kenneth E. Palmer

**Affiliations:** a Department of Microbiology and Immunology, School of Medicine, University of Louisville, Louisville, Kentucky, USA; b Department of Pharmacology and Toxicology, School of Medicine, University of Louisville, Louisville, Kentucky, USA; c Center for Predictive Medicine for Biodefense and Emerging Infectious Diseases, University of Louisville, Louisville, Kentucky, USA; The Ohio State University

**Keywords:** Griffithsin, Q-Griffithsin, antifungal, *Candida*, lectins, antifungal agents

## Abstract

There is a rising global incidence of *Candida* strains with high levels of resistance to fluconazole and other antifungal drugs, hence the need for novel antifungal treatment strategies. Here, we describe the first evidence of antifungal activity of Q-Griffithsin (Q-GRFT), a recombinant oxidation-resistant variant of Griffithsin, a marine red algal lectin with broad-spectrum antiviral activity. We demonstrated that Q-GRFT binds to α-mannan in the Candida albicans cell wall. We also observed that Q-GRFT binding disrupted cell wall integrity and induced reactive oxidative species (ROS) formation, resulting in cell death. Furthermore, we showed that Q-GRFT inhibited the growth of other *Candida* species C. glabrata, C. parapsilosis, and C. krusei and had modest activity against some strains of multi- and pandrug-resistant C. auris. We found that Q-GRFT induced differential expression of numerous genes involved in response to cell stress, including those responsible for neutralizing ROS production and cell cycle regulation. In conclusion, this novel antifungal activity suggests that Q-GRFT is potentially an ideal drug candidate and represents an alternative strategy for the prevention and treatment of candidiasis.

**IMPORTANCE** Fungal infections contribute to morbidity and mortality annually, and the number of organisms that are nonresponsive to the current available drug regimens are on the rise. There is a need to develop new agents to counter these infections and to add to the limited arsenal available to treat fungal infections. Our study has identified Q-GRFT, a broad-spectrum antiviral protein that harbors growth-inhibitory activity against several *Candida* strains, as a potential candidate for the prevention and treatment of fungal infections.

## INTRODUCTION

Long-term antifungal drug use, recurrent mucosal infections including chronic mucocutaneous candidiasis in human immunodeficiency virus (HIV) infection, and suboptimal mycotic-infection control have led to an increasing incidence of drug resistance (1, 2). In particular there are increasing reports of *Candida* species that demonstrate high resistance to fluconazole and other antifungal drugs (3). This problem is further complicated by the narrow spectrum and limited arsenal of currently available options, which include only polyenes, azoles, echinocandins, and flucytosine for invasive infections and allylamines for dermal mycoses (4–7). In addition, these current agents have been associated with toxicity and drug interactions, subsequently limiting their utility and contributing to the high morbidity and mortality in fungal infections (8, 9). Being eukaryotic organisms, *Candida* and fungi in general, unlike other microbes, such as bacteria, are closely related to humans and, hence, have a limited number of molecules that can be selected or identified as targets for the development of antimycotic agents (4, 6).

The latest Centers for Disease Control and Prevention (CDC) report, *Antibiotic Resistance Threats in the United States, 2019*, highlights the growing concern about fungal infections. In this report, drug-resistant Candida auris has been named an emerging urgent threat and drug-resistant *Candida* a serious threat, while azole-resistant Aspergillus fumigatus is on the watch list (10). This is in contrast to the previous 2013 report, which included fluconazole-resistant *Candida* as the only fungus that warranted attention as a serious threat then (11). Drug-resistant *Candida* species like Candida albicans, Candida parapsilosis, Candida krusei, and Candida glabrata account for more than 34,000 cases and 1,700 deaths annually (10, 12). Indeed, *Candida species* cause severe invasive infections that may often be difficult to differentiate from other systemic infections (13, 14). These include urinary tract infections, bloodstream infections, otitis, meningitis, wound infections, bone infections, myocarditis, and skin abscesses (15, 16). The difficulty involved in providing a definitive diagnosis for *Candida* infections, including C. auris, complicates early detection, further making treatment a challenge (17, 18). Typically, fungal infections are considered only when fever persists following antibiotic use (14). In addition, standard diagnostic approaches necessitate blood cultures, which are slow and often have sensitivities as low as 17% or 45% (14, 19, 20). Furthermore, drug-resistant C. auris has been associated with mortality rates ranging from 30% to 72% worldwide (13, 21–25), with some studies attributing 43% overall mortality to candidemia (26). Consequently, the difficulty in managing the treatment of patients with multidrug-resistant *Candida* species and pandrug-resistant isolates of Candida auris in the United States and globally (27–29) underscores the urgent need to develop new antifungal agents and therapeutic strategies.

Lectins are proteins that possess the ability to bind carbohydrates, often with high affinity and specificity (30). Griffithsin (GRFT) is an antiviral lectin originally derived from the red alga *Griffithsia* sp. and has been widely studied for its potent and broad-spectrum activity against human immunodeficiency virus (HIV) and other viruses (31–33). Native GRFT, a domain-swapped homodimer, binds to glycoproteins in the viral envelope and outer structure in a monosaccharide-dependent manner (30, 31, 34–37). GRFT’s antiviral properties are based on efficient and effective binding to oligosaccharides with mannose terminal branches (36). Additionally, GRFT binds to *N*-acetylglucosamine (GlcNAc) and glucose, albeit with lower affinities than to mannose (36). In the course of optimizing topical formulation strategies for GRFT-based microbicides, we discovered that a surface methionine residue at amino acid 78 was susceptible to oxidation and might therefore have a deleterious effect on product stability (38). We replaced this moiety with a glutamine (Q) residue and found that this molecule (Q-GRFT) has superior environmental stability with antiviral activity similar to that of GRFT and is hence more suitable for clinical development than the wild-type molecule. A rectal enema formulation of Q-GRFT designed to be a coitally dependent preexposure prophylaxis method was recently administered to volunteers in a first-in-humans clinical trial for prevention of HIV infection (ClinicalTrials.gov identifier NCT04032717) (39).

While investigating the mucosal interactions of Q-GRFT with gut microbiota, we discovered a novel, hitherto unreported antifungal activity of this lectin. Here, we present the findings of the impact of Q-GRFT on the growth of *Candida* spp. and identify a possible mechanism of antifungal activity *in vitro*. These findings support further exploration and development of Q-GRFT as an antifungal agent.

## RESULTS

### Effect of Q-GRFT on the growth of rectal microbiome (bacterial) components.

**FIG 1:**
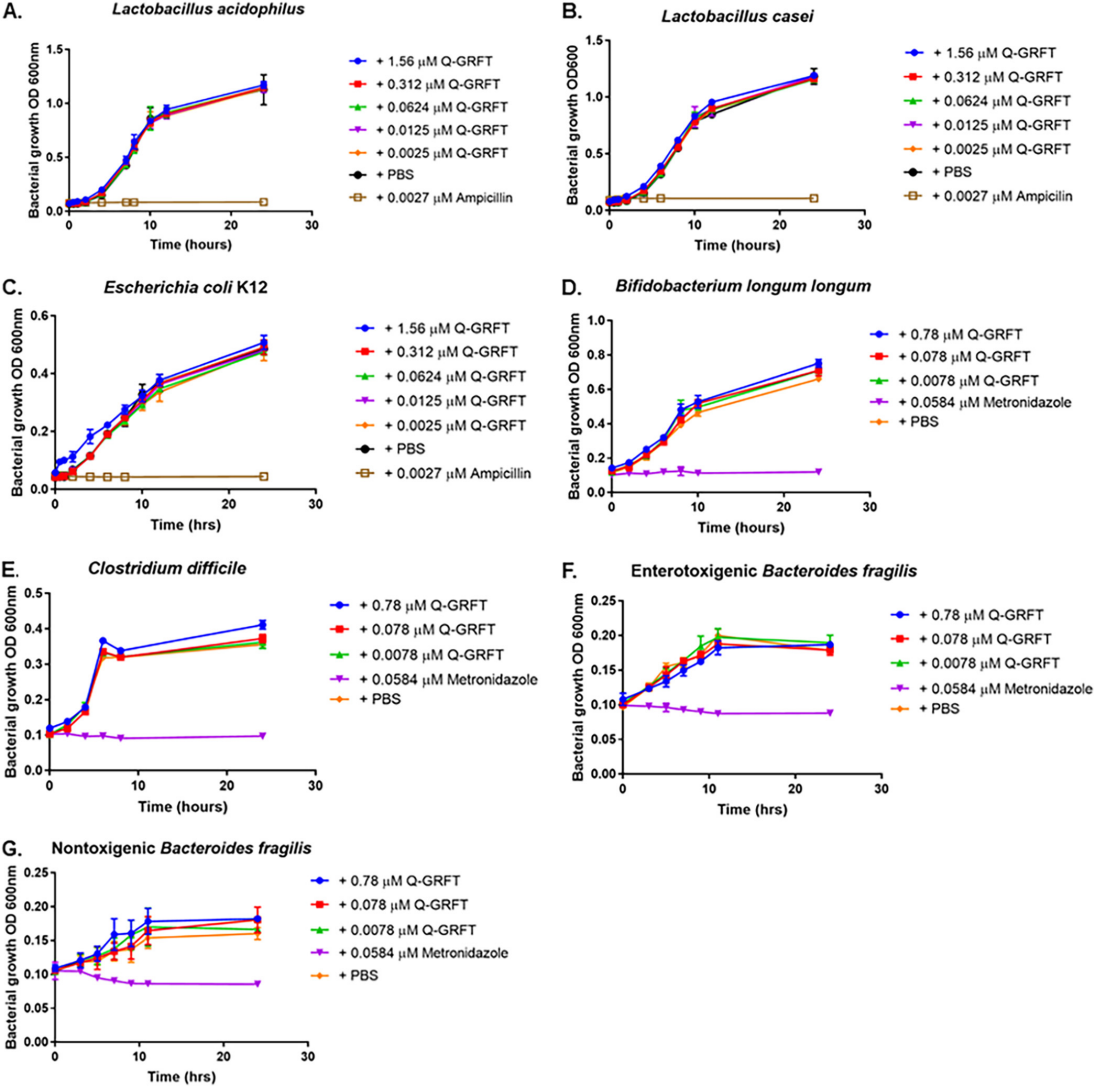
Effect of Q-GRFT on the growth of select bacterial species found in the human gut. (A) Lactobacillus acidophilus; (B) Lactobacillus casei; (C) Escherichia coli K-12; (D) Bifidobacterium longum subsp. *longum* ATCC 15707; (E) Clostridium difficile ATCC 51695-F2; (F) enterotoxigenic Bacteroides fragilis; and (G) nontoxigenic Bacteroides fragilis. Microbiome components were incubated with various concentrations of Q-GRFT, PBS vehicle, or control antibiotics in broth media, and growth monitored periodically by determining OD measurements using a plate reader. Data are mean values ± SD from at least 3 independent experiments.

While exploring the impacts on the proteome and microbiome when GRFT was applied topically as a rectal gel in nonhuman primates, Girard et al. (40) found that the lectin induced small but significant increases in the relative abundances of *Ruminococcaceae* NK4A214 and *Christensenellaceae* R-7, but the effect was not sustained at 7 days postapplication. No other significant effects were observed in Rhesus macaque rectal microbiota after application of the gel. Against this background, we investigated the effect of Q-GRFT on the growth of representative bacteria found in the human gut, including Lactobacillus acidophilus, Lactobacillus casei, Escherichia coli strain K-12, Bifidobacterium longum subsp. *longum* strain ATCC 15707, Clostridium difficile strain ATCC 51695-F2, enterotoxigenic Bacteroides fragilis, and nontoxigenic Bacteroides fragilis (Fig. 1). The selected bacterial components were incubated in the presence of various concentrations of Q-GRFT in broth media, and growth was established by determining optical density (OD) measurements over time using a plate reader. Q-GRFT had no detectable impact on the growth of any of the bacterial species tested.

We then tested Q-GRFT’s growth-inhibitory activity against select disease-causative pathogens for sexually transmitted infections, Chlamydia trachomatis and Neisseria gonorrhea. There was no detectable impact of Q-GRFT on the *in vitro* growth of Chlamydia trachomatis and Neisseria gonorrhea (Tables S1 and S2 and Fig. S1 in the supplemental material).

### Effect of Q-GRFT on the growth of Candida albicans.

**FIG 2:**
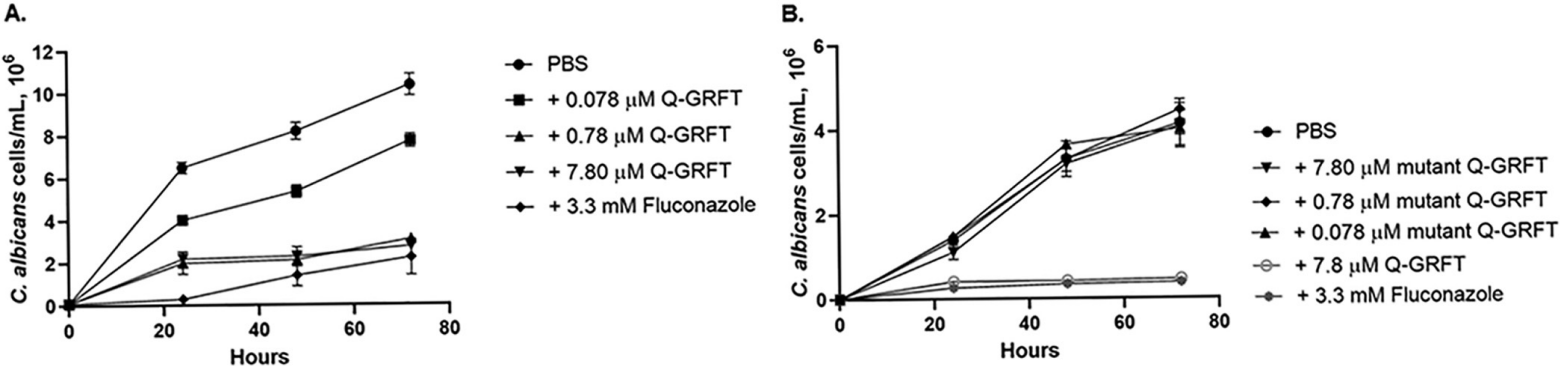
Effect of Q-GRFT on the growth of Candida albicans. (A) Candida albicans ATCC 32032 at a concentration of 1.0 × 10^5^ cells/ml was incubated with various concentrations of Q-GRFT and fluconazole control at 37°C in Sabouraud dextrose culture medium, and growth monitored at 24, 48, and 72 h. (B) C. albicans (1.0 × 10^4^ cells/ml) was incubated with different concentrations of the mutant Q-GRFT and Q-GRFT^lec neg^ (7.80 M and 0.78 M), with fluconazole (3.3 mM) and PBS as controls, at 37°C in Sabouraud dextrose culture medium. Growth was monitored at 24, 48, and 72 h. Fungal counts were performed using either a Bio-Rad TC10 automated cell counter or an ECHO Rebel hybrid microscope (RBLTEW31). Both experiments were performed in triplicate. Representative data (mean values ± SD) from at least 3 independent experiments are shown.

Since yeasts and fungi are universally present in gut microbial communities (41), we next investigated the impact of Q-GRFT on the growth of Candida albicans strain ATCC 32032, as a representative of the gut fungal community. Yeast counts were determined using a Bio-Rad TC10 automated cell counter (Bio-Rad Laboratories, Singapore) or an Echo Rebel hybrid microscope (RBLTEW31; Echo, San Diego, CA, USA). Following incubation with Candida albicans for 24, 48, and 72 h (Fig. 2A), Q-GRFT significantly inhibited fungal growth at all concentrations tested (7.80 μM, 0.78 μM, and 0.078 μM) (*P < *0.0001). Next, we sought to confirm Q-GRFT’s growth-inhibitory activity by incubating C. albicans with a Q-GRFT variant, Q-GRFT^lec neg^ (mutant Q-GRFT), that is devoid of its glycan binding ability. Incubation of C. albicans with various concentrations of Q-GRFT^lec neg^ for up to 72 h did not demonstrate any inhibitory impact on growth (Fig. 2B), suggesting a role of Q-GRFT’s binding in fungal inhibitory activity.

### Q-GRFT’s growth-inhibitory activity is dependent on the lectin’s binding to Candida albicans.

**FIG 3:**
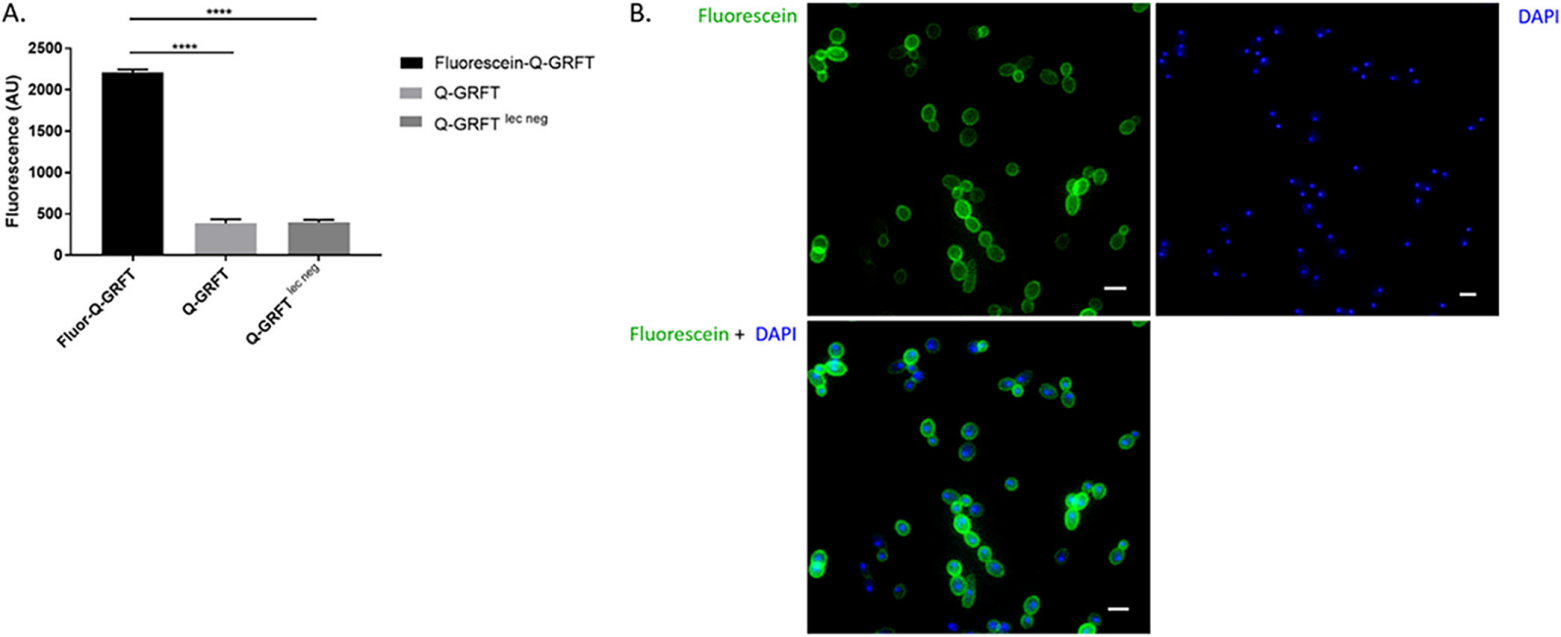
Q-GRFT binds to Candida albicans. (A) Fluorescence intensity of Candida albicans cultured with fluorescein–Q-GRFT, Q-GRFT, or Q-GRFT^lec neg^ at a lectin concentration of 7.8 μM. ****, *P* < 0.0001. (B) Fluorescence microscopy following culture of Candida albicans with fluorescein–Q-GRFT. Green fluorescence demonstrates localization to C. albicans cells. DAPI demonstrates DNA staining. Scale bars represent 3 μm. Experiments were performed in triplicate and repeated at least 3 times. The results shown are mean values ± SD (A) and representative images of the microscopy studies (B).

Since Q-GRFT inhibited the growth of C. albicans, we sought to confirm if this activity was dependent on Q-GRFT binding. C. albicans (1.0 × 10^5^ cells/ml) was cultured overnight with fluorescein-labeled Q-GRFT, unlabeled Q-GRFT, or fluorescein-labeled Q-GRFT^lec neg^. Cells were then centrifuged and washed, and the fluorescence intensity of the pellet determined. C. albicans incubated with fluorescein–Q-GRFT displayed the highest fluorescence intensity compared to the nonlabeled Q-GRFT (*P < *0.0001) and fluorescein–Q-GRFT^lec neg^ (*P < *0.0001) (Fig. 3A). To confirm lectin binding, C. albicans was cultured overnight with either fluorescein–Q-GRFT, Q-GRFT, or fluorescein–Q-GRFT^lec neg^ and the cells visualized using fluorescence microscopy. Green fluorescence was observed around yeast cells incubated with fluorescein–Q-GRFT, confirming Q-GRFT’s binding to C. albicans (Fig. 3B). No fluorescence was observed following incubation with fluorescein–Q-GRFT^lec neg^ (data not shown).

### Q-GRFT binds to Candida albicans cell wall component α-mannan but not chitin or β-glucan.

**FIG 4:**
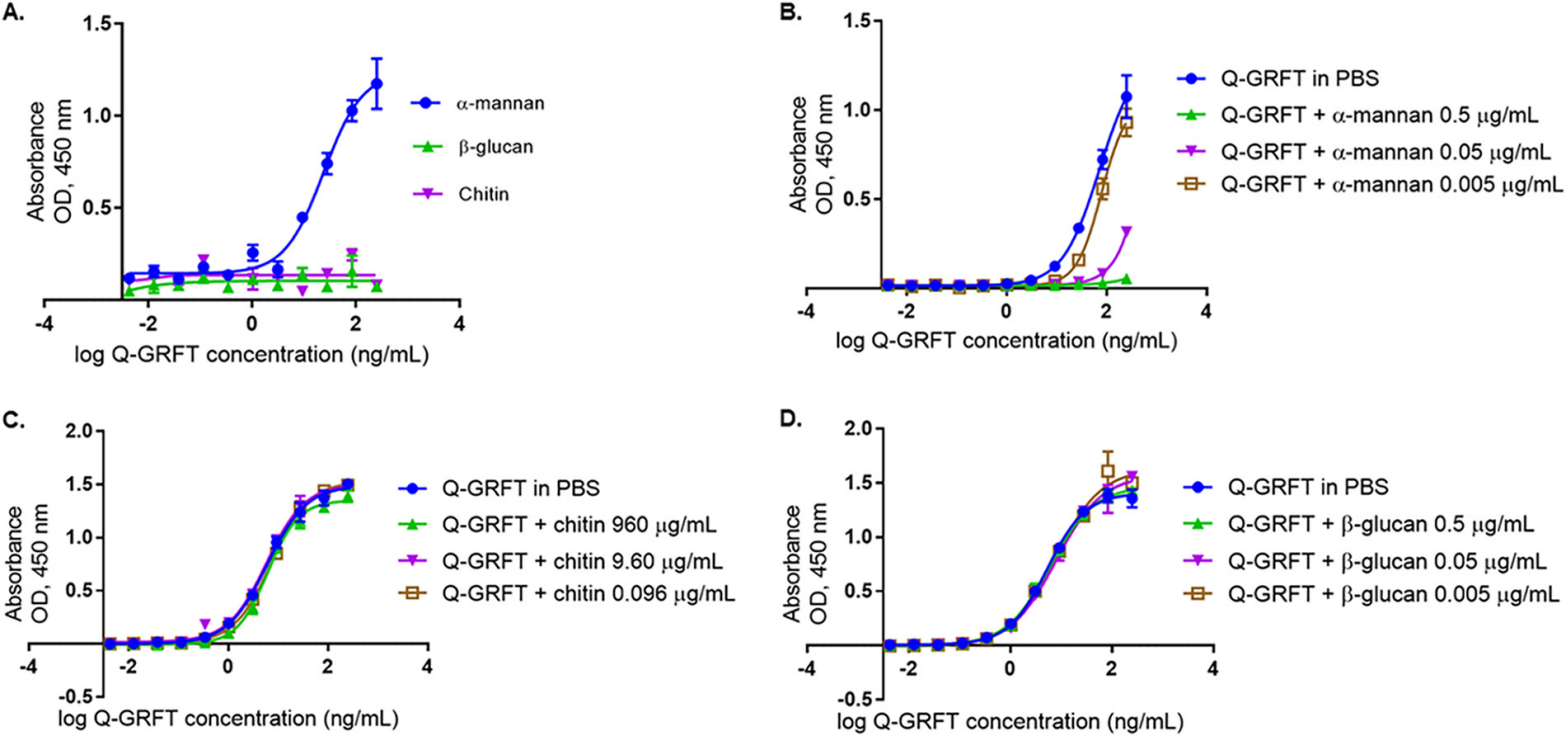
(A) Q-GRFT binds to Candida albicans cell wall component α-mannan but not chitin or β-glucan. An ELISA binding assay was performed, with the plate coated with 100 μl of α-mannan (20 μg/ml), chitin (2.0 mg/ml), or β-glucan (40 μg/ml). Q-GRFT’s binding to these components was then determined. (B) Unlike the low concentration (0.005 μg/ml), higher concentrations of α-mannan (0.5 μg/ml and 0.05 μg/ml) inhibited Q-GRFT’s binding to gp120 in a competition ELISA. (C and D) Q-GRFT was incubated with free (unbound) α-mannan and its ability to bind gp120 determined. When incubated with free (unbound) chitin (C) and β-glucan (D), Q-GRFT’s binding to gp120 was not inhibited in a competition ELISA. Experiments were performed in triplicate and repeated at least 3 times. Representative data (mean values ± SD) from the experiments are shown.

In order to better understand the binding, we investigated the C. albicans cell wall components to which Q-GRFT binds. Since the lectin bound to C. albicans, we hypothesized that Q-GRFT likely binds to chitin, glucans, or mannans, which are predominant core components of the fungal cell wall (42, 43). In addition, chitin is a polymer of *N*-acetyl-d-glucosamine (d-GlcNAc) (44), while mannan is an *N*-glycosylated polysaccharide with oligomannose side chains and branch chain mannose residues (45). Furthermore, β-glucans are composed of linking glucose units (42). These structures, therefore, provide likely binding targets for lectins, including Q-GRFT (36). Using enzyme-linked immunosorbent assay (ELISA) binding assays (Fig. 4A), we determined the ability of Q-GRFT to bind to plate-immobilized antigens α-mannan, β-glucan, and chitin. Q-GRFT bound to α-mannan with a 50% effective concentration (EC_50_) of 23.47 ng/ml (95% confidence interval [CI], 17.63 to 35.25 ng/ml). Q-GRFT did not bind to chitin or β-glucan. Because Q-GRFT binds to gp120, we next sought to determine if the lectin’s binding to the glycoprotein was inhibited in the presence of free and unbound cell wall component α-mannan, chitin, or β-glucan in a competition assay. Additionally, as antigens attach to the solid interface in the ELISA, they are likely to denature and/or undergo conformational changes that may affect their interaction with other biomolecules (46). Therefore, Q-GRFT was incubated with free α-mannan, chitin, or β-glucan prior to determining its binding to gp120. Q-GRFT’s binding to gp120 was inhibited in the presence of high concentrations of α-mannan (Fig. 4B): the EC_50_ in phosphate-buffered saline (PBS) was 72.46 ng/ml (95% CI, 55.17 to 115.1 ng/ml), while in α-mannan at 0.005 μg/ml, the EC_50_ was 79.35 ng/ml (95% CI, 69.19 to 97.85 ng/ml). However, Q-GRFT’s binding to gp120 was not inhibited in the presence of either free chitin (Fig. 4C) or β-glucan (Fig. 4D). For the free-chitin-binding assay, the EC_50_ of Q-GRFT in PBS was 6.017 ng/ml (95% CI, 5.227 to 6.992 ng/ml), the EC_50_ in 960 μg/ml chitin was 6.176 ng/ml (95% CI, 5.451 to 7.043 ng/ml), the EC_50_ in 9.60 μg/ml chitin was 5.785 ng/ml (95% CI, 4.851 to 6.975), and the EC_50_ in 0.0960 μg/ml chitin was 7.295 ng/ml (95% CI, 6.732 to 7.921). For the free–β-glucan-binding competition assay, the EC_50_ of Q-GRFT in PBS was 5.319 ng/ml (95% CI, 4.734 to 5.991 ng/ml), the EC_50_ in 0.5 μg/ml β-glucan was 6.012 ng/ml (95% CI, 5.366 to 6.77 ng/ml), the EC_50_ in 0.05 μg/ml β-glucan was 7.917 ng/ml (95% CI, 6.505 to 9.901 ng/ml), and the EC_50_ in 0.005 μg/ml β-glucan was 7.768 ng/ml (95% CI, 5.896 to 10.58 ng/ml). The results from these binding studies confirmed that Q-GRFT binds to α-mannan but not chitin or β-glucan in the C. albicans cell wall.

### Effect of Q-GRFT on Candida albicans’ cell structure, integrity, and oxidative status.

**FIG 5:**
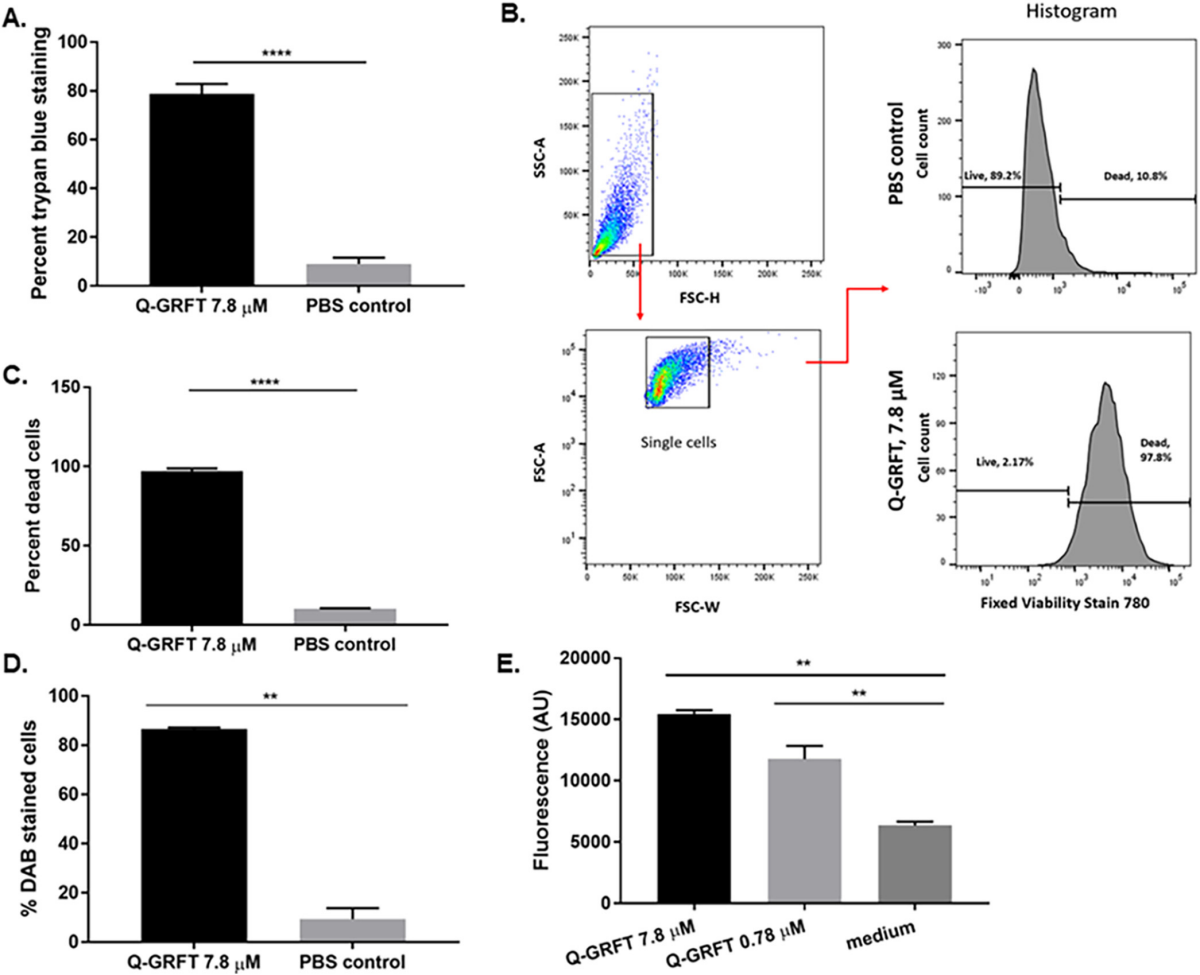
Effects of Q-GRFT on Candida albicans’ cell integrity and oxidative status. (A) Yeast cells were incubated in the absence or presence of Q-GRFT (7.8 μM) overnight at 37°C in Sabouraud dextrose medium, followed by staining with trypan blue to detect nonviable cells. Data are representative of results from at least 3 experiments with 3 biological replicates (mean values ± SD, *P < *0.0001). (B) C. albicans (1.0 × 10^5^ cells/ml) was incubated overnight with either Q-GRFT 7.8 μM or PBS control, and cell viability determined using flow cytometry. Dead cells were identified as those that were able to take up the dye. Representative gating strategy for both Q-GRFT-treated and PBS control-treated cells is presented. SSC, side scatter; FSC, forward scatter; A, area; H, height; W, width. (C) Quantification of the percentages of dead cells obtained from flow cytometry measurements for Q-GRFT-treated and PBS-treated C. albicans in the experiment whose results are shown in panel B. Experiments were performed three times, and representative data (mean values ± SD, *P < *0.0001) are shown. (D) C. albicans cells were incubated with Q-GRFT (7.8 μM) or PBS overnight, followed by incubation in the presence of 0.5 mg/ml DAB for 2 h to detect hydrogen peroxide. Quantification of the proportion (percentage) of DAB-stained cells following either Q-GRFT or PBS treatment is shown (mean ± SD, *P* = 0.0017). (E) C. albicans was incubated overnight with Q-GRFT (7.8 and 0.78 μM) or medium only at 37°C. Cells were harvested, and 3.2 × 10^6^ cells per treatment used for direct fluorescence experiments using the H_2_DCF-DA assay to determine reactive oxygen species (ROS) levels in the cells. Experiments were performed in triplicate, and representative data (mean ± SD) are shown (Q-GRFT 7.8 μM, *P = *0.0015, and Q-GRFT 0.78 μM, *P = *0.0068).

Treatment of C. albicans with helianthus annus (Helja) lectin has been shown to alter membrane permeability and induce intracellular formation of oxidative species (47). Therefore, we hypothesized that Q-GRFT lectin may act similarly, altering fungal cell wall permeability and inducing the expression of intracellular reactive oxygen species (ROS), with subsequent cellular injury and/or damage, leading to cell death. To demonstrate the impact on cell wall integrity, C. albicans was incubated with 7.8 μM Q-GRFT overnight at 37°C, followed by trypan blue staining, with the dye uptake demonstrating breached cell wall/disrupted membrane integrity and penetration into dead/nonviable cells. Compared to the staining in the PBS control, Q-GRFT treatment resulted in significant intracellular blue staining, indicative of nonviable cells with impaired cell wall integrity (*P < *0.0001) (Fig. 5A). To further confirm disruption of cell wall and membrane integrity, we performed live/dead cell viability staining of 7.8 μM Q-GRFT- and PBS control-treated C. albicans cells, with measurements determined by flow cytometry (Fig. 5B and C). These cells were incubated at 37°C overnight under the respective treatment conditions. Figure 5B shows a representative image of the gating strategies for both Q-GRFT and PBS treatments. Fixable viability stain 780 (FVS780)-positive cells were considered dead cells with disrupted membrane integrity. Q-GRFT treatment resulted in a significantly higher proportion of dead cells than the PBS control treatment (*P < *0.0001) (Fig. 5C).

To investigate the induction of ROS by Q-GRFT, C. albicans cells were incubated overnight with 7.8 μM Q-GRFT followed by incubation with DAB (3,3′-diaminobenzidine) for 2 h. In the presence of peroxides, DAB is oxidized to an insoluble brown precipitate that is visualized within cells using optical microscopy. Compared to the PBS vehicle-treated control, a significantly large proportion of Candida albicans cells incubated with Q-GRFT developed a brown intracellular precipitate. Quantification of this effect revealed that the presence of peroxides was significantly higher (*P < *0.0017) following Q-GRFT treatment than with PBS control (Fig. 5D). To confirm the presence of ROS, we used the H_2_DCF-DA (2′,7′-dichlorodihydrofluorescein diacetate) assay technique to profile the oxidative status of Q-GRFT-treated cells and PBS vehicle-treated control cells. This assay is dependent on the ability of cellular esterase to cleave acetate groups on H_2_DCF-DA, releasing an intermediate H_2_DCF product that reacts with ROS, forming fluorescent DCF (2′,7′-dichlorofluorescein) (48). Compared with PBS vehicle-treated control cells, Q-GRFT treatment was associated with higher fluorescence activity (*P < *0.002 and *P < *0.006 for 7.8 μM and 0.78 μM, respectively) following the H_2_DCF assay (Fig. 5E). Centrifugation of cells during preparation for the H_2_DCF assay may induce ROS formation, accounting for the low levels observed in the negative-control (PBS vehicle-treated) cells (48). It is likely that differential Q-GRFT treatment with various drug concentrations elicits dissimilar levels of cellular stress, accounting for the differences in ROS formation for the 7.8 μM and 0.78 μM Q-GRFT treatments.

**FIG 6:**
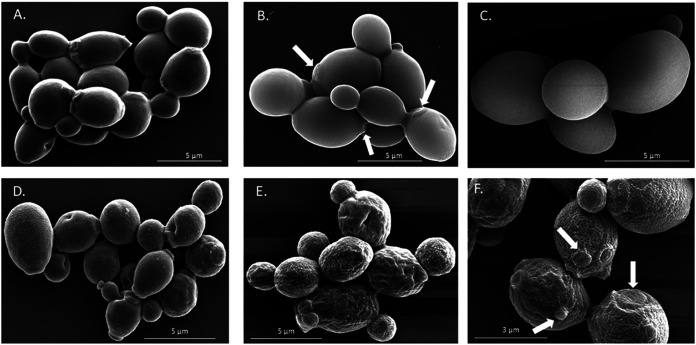
C. albicans surface phenotype following treatment with Q-GRFT. C. albicans was grown overnight in Sabouraud dextrose medium in the presence of either PBS vehicle (A to C) or 7.8 μM Q-GRFT (D and E) at 37°C. Cells were then observed by SEM. (A) Budding morphology of C. albicans. Note the spherical to oval appearance of cells. Magnification, ×7,500. (B) Cells with smooth surface, polar budding, and bud scars (arrows). Magnification, ×10,000. (C) Note the smooth cell surface and absence of any ‘nonpolar’ bud scars. Magnification, ×12,500. (D) Cells with spherical to circular shape with rough surface. Magnification, ×7500. (E) Note the rough surface and uniform indentations with a desiccated and wrinkled appearance. Magnification, ×10,000. (F) Note the loss of polar budding, more circular appearance, and cells with multiple bud scars (arrows). Magnification, ×17,500.

To further evaluate any structural changes to C. albicans following treatment with Q-GRFT, high-resolution scanning electron microscopy (SEM) was performed for both Q-GRFT-treated cells and PBS vehicle-treated control cells (Fig. 6). Yeast cells were treated with either 7.8 μM Q-GRFT or PBS vehicle and incubated overnight at 37°C prior to imaging. The vehicle-treated control cells demonstrated a normal budding pattern and were predominantly spherical to oval in shape (Fig. 6A), with polar buds and bud scars (Fig. 6B) and smooth edges and surfaces (Fig. 6C). Q-GRFT-treated cells were spherical to circular (Fig. 6D and E), rough in appearance, demonstrated desiccated and wrinkled surfaces with uniform indentations (Fig. 6E and F), and had a loss of polar budding (Fig. 6F).

### Impact of Q-GRFT on the growth of non-albicans Candida species.

**FIG 7:**
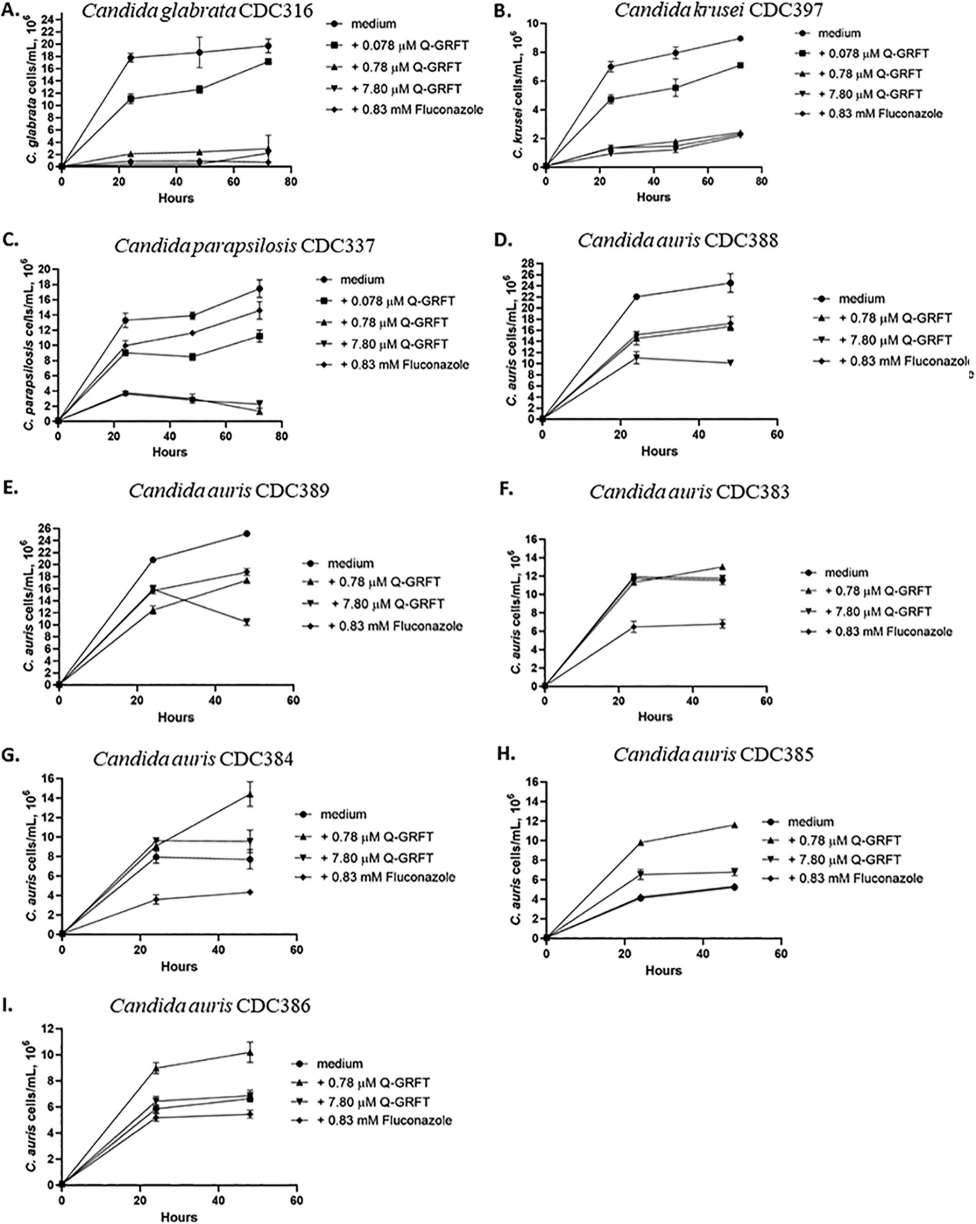
Impact of Q-GRFT on growth of non-*albicans Candida* species at 24, 48, and 72 h. (A) Candida glabrata CDC316; (B) Candida krusei CDC397; (C) Candida parapsilosis CDC337 and multidrug-resistant Candida auris strains; (D) Candida auris CDC389; (E) Candida auris CDC389; (F) Candida auris CDC383; (G) Candida auris CDC384; (H) Candida auris CDC385; and (I) Candida auris CDC386. *Candida* strains at a concentration of 1.0 × 10^5^ cell/ml were incubated with different concentrations of Q-GRFT and fluconazole control at 37°C in Sabouraud dextrose culture media. Growth was monitored up to 72 h. Fungal counts were performed using either a Bio-Rad TC10 automated cell counter or an ECHO Rebel hybrid microscope (RBLTEW31). Data represent mean values ± SD from 3 independent experiments.

Given the recent increase in *Candida* species resistance to antifungal drugs (3), we next investigated the impact of Q-GRFT on the growth of human-pathogenic non-albicans Candida species, including the multidrug-resistant (MDR) Candida auris. Most of the C. auris strains isolated from patients and identified in the United States belong to clades originating from South America and South Asia (49). We therefore tested Q-GRFT’s growth-inhibitory activity on representative isolates from these and other clades, including strains C. auris CDC388 and C. auris CDC389 from a South Asian clade, C. auris CDC385 and C. auris CDC386 from a South American clade, and C. auris CDC383 and C. auris CDC384 from an African clade. The other non-albicans Candida species tested included Candida glabrata CDC316, Candida krusei CDC397, and Candida parapsilosis CDC337. Q-GRFT was incubated with Candida glabrata, Candida krusei, and Candida parapsilosis at 37°C and growth monitored periodically using a cell counter, at 24, 48, and 72 h. Compared to the growth in the medium control, Q-GRFT significantly inhibited the growth of all the species tested, with the greatest effect demonstrated with the 7.80 μM lectin concentration (*P < *0.0001 for all species and concentrations tested) (Fig. 7A to C). When incubated with Candida auris, Q-GRFT significantly inhibited the growth of strains Candida auris CDC388 and Candida auris CDC389 (*P < *0.0001 for both lectin concentrations tested, 0.78 μM and 7.8 μM) (Fig. 7D and E). There was no observable impact on the growth of strains Candida auris CDC383 (Fig. 7F), Candida auris CDC384 (Fig. 7G), Candida auris CDC385 (Fig. 7H), and Candida auris CDC386 (Fig. 7I).

### MICs for Q-GRFT against *Candida* spp.

Starting with a maximum concentration of 95 μg/ml, MICs were determined for Q-GRFT’s activity against different *Candida* strains and isolates and are summarized in Table 1. The MIC_50_s for Q-GRFT against C. albicans, C. glabrata, C. parapsilosis, C. krusei, C. auris CDC388, and C. auris CDC389 were 6, 95, 24, 95, 48, and 95 μg/ml, respectively, while the MIC_90_ against C. albicans, C. parapsilosis, and C. auris CDC389 was 95 μg/ml for all isolates.

**TABLE 1:** MIC_50_s and MIC_90_s for Q-GRFT against different *Candida* species

*Candida* isolate	Value (μg/ml) for:	
	MIC_50_	MIC_90_
Candida albicans ATCC 32020	6	95
Candida glabrata CDC316	95	
Candida parapsilosis CDC337	24	95
Candida krusei CDC397	95	
Candida auris CDC388	48	95
Candida auris CDC389	95	

### Q-GRFT treatment alters the expression of multiple genes important in Candida albicans growth.

**FIG 8:**
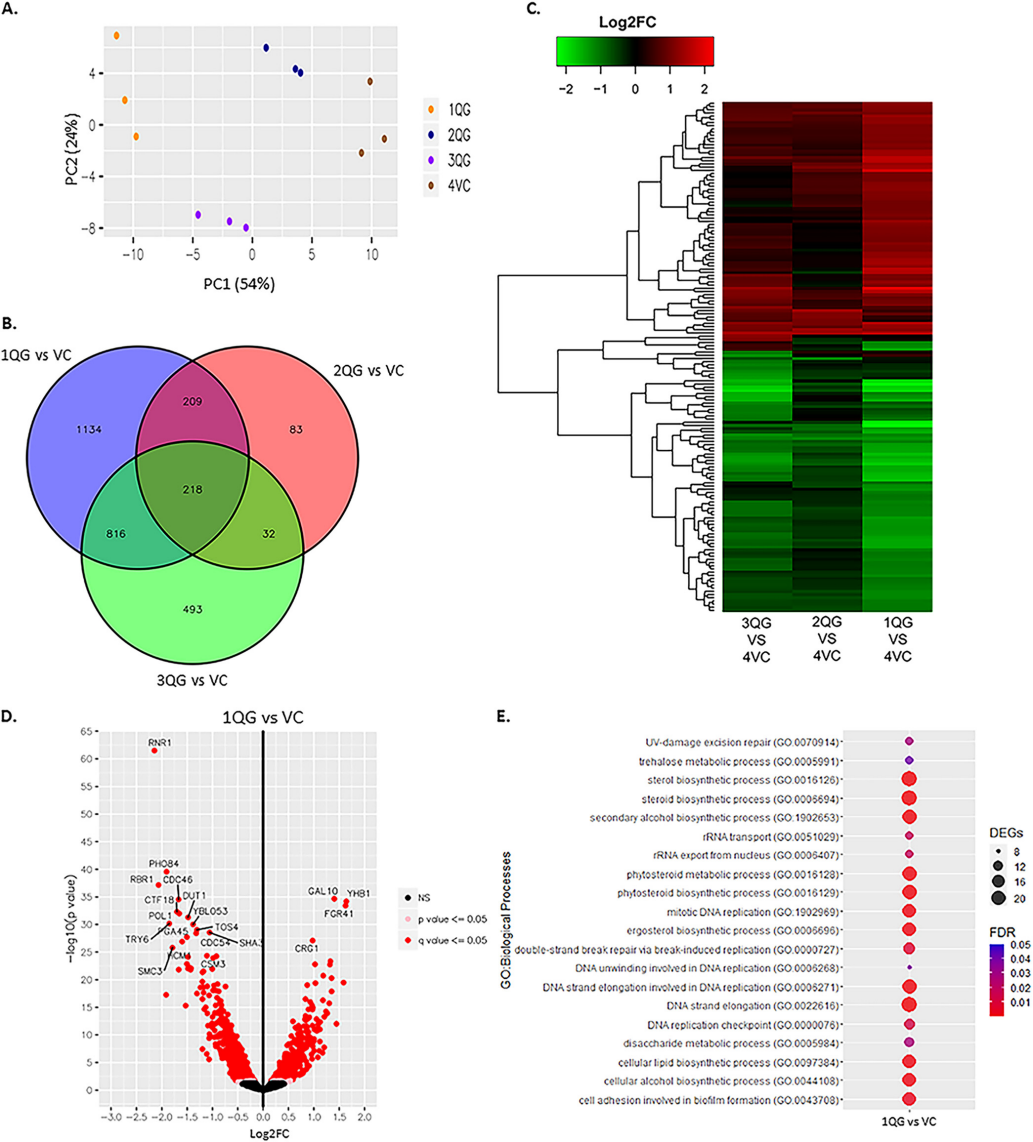
C. albicans RNA expression profile following treatment with Q-GRFT. C. albicans at a concentration of 1.0 × 10^5^ cells/ml was incubated overnight at 37°C in Sabouraud dextrose medium with 7.8 μM (1QG), 0.78 μM (2QG), or 7.8 μM Q-GRFT^lec neg^ (3QG) or control medium (vehicle control [VC]). RNA was isolated and sequencing performed to determine expression under the different treatment conditions. Experiments were performed in triplicate. (A) Principal component analysis (PCA) plot performed on normalized read counts for all samples. Similar colors represent cells that were subjected to a similar treatment. (B) Venn diagram showing shared differentially expressed genes for all DEGs. The intersection of the three circles represents overlapping DEGs among the three treatments. (C) Cluster analysis of DEGs for genes showing differential expression of |log_2_FC| ≥ 2 (FC, fold change) for the comparison following different treatments. Red represents upregulation; green represents downregulation. (D) Volcano plot to examine the log_2_ fold change of DEGs with 1QG treatment, for a significance level of *P* <* *0.05 and adjusted *P* value (*Q*) of <0.05. Log_2_ fold change on the *x* axis is plotted against −log_10_(*P* value) on the *y* axis. (E) Enriched GO functions of DEGs. Top 20 enriched biological processes with the adjusted *P* values (false discovery rate [FDR]) for the 1QG versus VC treatment groups.

To better understand the impact of Q-GRFT on C. albicans growth and metabolism, we sought to determine the differential mRNA expression profile upon Q-GRFT treatment. C. albicans was treated overnight with 7.8 μM Q-GRFT (1QG), 0.78 μM Q-GRFT (2QG), or 7.8 μM Q-GRFT^lec neg^ (3QG), and comparisons were made with the PBS vehicle-treated controls (VC) following RNA isolation and sequencing. The principal-component analysis (PCA) results showed that the experimental groups clearly had good separation based on treatment status (Fig. 8A). Differential expression analysis was performed for the different treatments using DESeq2. Compared to the gene expression in VC, Venn diagram analysis showing shared differentially expressed genes (DEGs) for all DEGs demonstrated that totals of 2,377, 542, and 1,559 genes were differentially expressed following 1QG, 2QG, and 3QG treatment, respectively (Fig. 8B). Of the 2,377 DEGs in the 1QG group, 209 were uniquely expressed by both the 1QG and 2QG groups, while 816 were uniquely expressed by both the 1QG and 3QG groups. Additionally, of the 542 DEGs in the 2QG group, 32 were uniquely expressed by both the 2QG and 3QG groups. Two hundred eighteen DEGs were similar among all 3 treatment groups. Treatment in the 1QG group resulted in 1,134 DEGs unique to only this group, while 83 and 493 DEGs were uniquely expressed by the 2QG and 3QG groups, respectively.

Analysis of upregulated DEGs (Fig. S2A) identified 1,168, 308, and 734 genes whose expression was elevated in the 1QG, 2QG, and 3QG groups, respectively. Of the 1,168 in the 1QG group, 131 upregulated genes were uniquely expressed by both 1QG and 2QG, while 344 genes were uniquely upregulated by both 1QG and 3QG groups. In addition, 16 DEGs were uniquely upregulated by both the 2QG and 3QG treatment groups. One hundred eight upregulated DEGs were similar following treatment in all 3 groups. Among the downregulated DEGs (Fig. S2B), the expression of a total of 1,209 genes was decreased in the 1QG group, while 234 and 825 genes were decreased in the 2QG and 3QG groups, respectively. Of the 1,209 DEGs in 1QG, 77 genes were uniquely downregulated in the 1QG and 2QG groups only, while the expression of 457 genes was uniquely decreased in the 1QG and 3QG groups only. Of the 234 downregulated DEGs in the 2QG group, 6 genes were uniquely decreased in the 2QG and 3QG groups only. Among all groups, a total of 108 similar genes were downregulated across all treatments. Cluster analysis of the DEGs for all groups demonstrated significant differences upon treatment (Fig. 8C and Fig. S2C). Further analysis of differentially expressed genes in the 1QG treatment group demonstrated significantly abundant elevations of genes involved in carbohydrate metabolism (*GAL10*), nitric oxide metabolism (*YHB1*), filamentous growth (*FGR41*), and DNA and protein synthesis, among other genes (adjusted *P* value of < 0.05). Genes showing reduced expression included those involved in RNA synthesis (*RNR1*), filamentation (*RBR1*), and phosphate ion transport (*PHO84*), among others (adjusted *P* value of < 0.05) (Fig. 8D). Kyoto Encyclopedia of Genes and Genomes (KEGG) pathway analysis indicated that upregulated DEGs following 1QG treatment were highly associated with metabolic pathways, ribosome, metabolite biosynthesis, cell cycle and DNA repair, and response to oxidative stress (like *SOD3*), among others (Table S3). The top 20 downregulated DEGs for 1QG versus VC treatment are shown in Table S4. Low-dose treatment (2QG) demonstrated elevation of genes required to maintain mitochondrial respiration during stressful periods (*AOX1*, *AOX2*, and *MRF1*), glutamate metabolism (*GAD1*), and cell adhesion and virulence (*PGA22*), among others. Conversely, genes responsible for meiotic division (*MCD1* and *CCN1*), iron metabolism (*HMX1*), and filamentous growth and hypha formation during cell stress (*SHA3* and *GIN4*), among others, were significantly decreased (Fig. S2D).

KEGG pathway analysis indicated that following 2QG treatment, upregulated DEGs were highly associated with glycolysis/gluconeogenesis, carbon metabolism, biosynthesis of secondary metabolites, cell cycle, and DNA replication and repair, among others (Table S5). The top 20 downregulated DEGs for 2QG versus VC treatment are shown in Table S6. Treatment with Q-GRFT^lec neg^ resulted in decreased expression of genes involved in iron metabolism (*FRE7* and *FRE30*), copper homeostasis (*CTR1*), biofilm formation, and RNA synthesis (*RNR1*) (Fig. S2E). Upon treatment with Q-GRFT^lec neg^, KEGG pathway analysis revealed upregulation of genes involved with metabolic pathways, cell cycle, meiosis, and homologous recombination (Table S7). Interestingly, genes expressed in response to oxidative stress (*GPX2* and *SOD3*) were among those downregulated with Q-GRFT^lec neg^ treatment. The top 20 downregulated DEGs for 3QG versus VC treatment are shown in Table S8. The PANTHER Classification System (50) was used to identify enriched Gene Ontology (GO) biological processes for each set of differentially expressed genes. The results revealed that Q-GRFT treatment (1QG) significantly enriched biological processes involved in DNA damage repair, DNA replication, sterol and steroid biosynthesis, and disaccharide metabolism (Fig. 8E). Similarly, 2QG treatment enriched processes involved in DNA and RNA metabolism, carbohydrate metabolism including glucose, galactose and pyruvate, and amino acid catabolism (Fig. S3A). Q-GRFT^lec neg^ treatment enriched processes involved in DNA formation and elongation, cell adhesion, and biofilm formation (Fig. S3B), processes likely to promote and maintain cell growth. Given the difference in ROS formation between 7.8 μM and 0.78 μM Q-GRFT treatments demonstrated by the results shown in Fig. 5C, it is likely that the impact of the lectin on C. albicans’ gene expression profile is concentration dependent. This may partly explain the differences in RNA expression profiles observed upon treatment with high- and low-dose lectin concentrations. Moreover, overall, as observed with RNA expression, similar biologic processes were impacted following both treatments.

## DISCUSSION

Our findings reveal that Q-GRFT inhibits the growth of C. albicans and other *Candida* species C. glabrata, C. parapsilosis, and C. krusei, with modest activity against some strains of C. auris *in vitro.* We observed no demonstrable impact on the growth of select bacterial species commonly found in the gut. Similarly, Q-GRFT had no measurable effect on the growth of Neisseria gonorrhea and Chlamydia trachomatis, the causative pathogens for common sexually transmitted infections.

The gut microbiome plays a role in maintaining health, with dysbiosis implicated in promoting inflammation and disease (51, 52). Because Q-GRFT is currently being developed as a topical rectal microbicide (39), activity in the gut lumen and interaction with gut and rectal microbiome components is inevitable. In our previous studies (40), we found that repeated rectal treatment of macaques with Q-GRFT gels did not result in any detrimental changes in the resident bacterial microbiome compared with the baseline population. Our assessment of Q-GRFT’s impact on the growth of select bacteria cultured *in vitro* confirmed results by Girard et al. (40), as the lectin did not inhibit the growth of any species tested.

Like bacteria, yeast and fungi are components of the microbiome, but their role in the microbial gut community dynamics is not yet well described. Dysbiosis and overgrowth of fungal species results in a shift in the diversity and richness in group members, with subsequent inflammation (53, 54). *Candida* species infections pose a major health concern in both immunocompromised patients and immunocompetent individuals (55). Given the need to develop new antifungal agents and the increasing burden of disease posed by fungus and yeast infections, several other groups have investigated how natural-product lectins interact with human-pathogenic fungi. Sunflower Helianthus annuus lectin decreased C. albicans survival by impairing cell wall integrity, induced hydrogen peroxide formation, and inhibited the yeast-to-filamentous fungus morphological transition responsible for virulence. Helianthus annuus lectin also impaired fungal cell adherence to surfaces and interfered with biofilm development, resulting in a reduced coverage area (47). By binding to glucose, mannose, and *N*-acetylglucosamine in the fungal cell wall, other lectins have exhibited antifungal activity, with various degrees of growth inhibition. Lectin TEL (from Talisa esculenta fruit) inhibited Saccharomyces cerevisiae, Fusarium moniliforme, and Colletotrichum lindemuthianum growth, while AML (from Annona muricata soursop fruit), which binds glucose and mannose, inhibited Fusarium solani, Fusarium oxysporum, and Colletotrichum musae growth. These lectins inhibited fungal spore germination and mycelium growth. They also altered the synthesis of chitin, interfering with cell wall deposition and formation (56). Lectins DvioL, DRL, and ConBr are extracts from Dioclea violacea, Dioclea rostrata, and Canavalia brasiliensis legumes, respectively. These extracts bind glucose and mannose and have demonstrated antifungal activity against yeasts isolated from vaginal secretions, including Candida guilliermondii, Candida shehatae, Candida membranifaciens, and Kloeckera apiculata (56–59). Lectins produced by *Lactobacillus* spp. have been shown to bind to C. albicans and to inhibit biofilm formation (60). We determined the impact of Q-GRFT on the growth of C. albicans as a representative of the mycobiome in the gut and colon. We found that Q-GRFT bound to α-mannan in the fungal cell wall and inhibited the growth of C. albicans. We also showed that antifungal activity was dependent on lectin binding, since Q-GRFT^lec neg^ neither bound nor inhibited C. albicans growth. We demonstrated that the lectin impaired cell wall integrity by increasing permeability and induced the formation of ROS within cells.

Q-GRFT exposure to C. albicans may result in an osmotic imbalance caused by lectin-mediated disruption in cell wall integrity. We observed that Q-GRFT induced marked alterations in the physical appearance of C. albicans, with cells demonstrating a marked shriveled appearance and collapsed structure with surface indentations. Q-GRFT-treated cells also demonstrated increased attempts at budding, with multiple bud scars and a loss of the normal polar budding orientation. This is indicative that Q-GRFT-induced changes affect normal cell division. The loss of polar budding and multiple bud scars may be an attempt by C. albicans to divide multiple times to escape stress-induced conditions (61). This failed attempt is likely compounded by the downregulation of the *Tos4* gene that was observed following Q-GRFT treatment. The *Tos4* gene regulates the G_1_/S cell cycle phase, promoting cell division (62). This downregulation, together with other intracellular injury processes, would result in failure to complete cell division and subsequently lead to cell death. Additionally, in response to oxidative stress, C. albicans expresses antioxidant genes to neutralize and escape stress, including genes encoding superoxide dismutase (*SOD*), glutathione peroxidase (*GPX2*), thioredoxin (*TRX*), and thioredoxin reductase (*TRR*) (63). Q-GRFT treatment was associated with upregulation of *SOD*, while cells treated with the nonbinding Q-GRFT^lec neg^ exhibited downregulation of GPX2 and TRR1. Because we used *in vitro* assays to demonstrate growth inhibition, there is a likelihood that cells incubated with Q-GRFT^lec neg^ underwent somewhat stressful growth conditions, given the volume and space limitations with this assay. When cells grow without inhibition in medium, they will reach a critical mass when they start to compete for nutrients within the restricted space. This has the potential to induce metabolic responses within cells to escape these stressful conditions. However, given the clear differential expression of genes following Q-GRFT treatment in comparison to the gene expression in Q-GRFT^lec neg^-treated cells, it is evident that the lectin does impact multiple metabolic pathways within C. albicans upon treatment. Cell cycle arrest, disaccharide metabolism, biofilm formation, and DNA strand elongation were among the upregulated pathways following Q-GRFT^lec neg^ treatment. Similarly, Q-GRFT treatment demonstrated upregulated stress response pathways, including monosaccharide, glucose, galactose and amino acid metabolism, biofilm formation, and DNA replication, among others.

Here, we have established that Q-GRFT demonstrates potent inhibitory activity against non-albicans Candida species of clinical importance, i.e., Candida glabrata, Candida krusei, and Candida parapsilosis. Interestingly, C. krusei has been described to harbor innate resistance against fluconazole (64, 65), while azole resistance is increasingly being documented for C. glabrata and C. parapsilosis. In addition, our study established that Q-GRFT demonstrates growth inhibition of Candida auris CDC388 and Candida auris CDC389, strains belonging to the South Asian clade, one of those that are dominant in the United States (49). However, the growth of strains Candida auris CDC383, Candida auris CDC384, Candida auris CDC385, and Candida auris CDC386 was not impacted following incubation with Q-GRFT. C. auris exhibits multidrug resistance, and pandrug-resistant strains have recently been identified (17, 27, 66–68). *N*- and *O*-linked mannans with α-1,2-, α-1,3-, α-1,6-, and β-1,2-linked mannose sugar residues make up the structure of mannoproteins in the *Candida* cell wall (69). Because we have identified α-mannan as a binding ligand for Q-GRFT (Fig. 4), it is likely that the highly diverse composition of mannan structures and mannosyl residues in different *Candida* species (70) contributes to the differential response to cell treatment and variations in MICs observed in our study. Mannan in C. albicans is comprised of branching *N*-linked polysaccharide units and short-chain *O*-linked mannan oligosaccharides. Additionally, the *N*-linked mannan is made up of a long-chain α-1,6-linked mannose backbone that bridges with oligomannose side chains predominantly consisting of α-1,2-, α-1,3-, and β-1,2-linked mannose residues with sparse phosphate groups (69–71). The mannan in C. glabrata is comprised of small branches with minimal α-mannan content and one or two β-1,2-linked mannose residues (69, 70, 72). A long chain of α-1,2-linked mannose subunits with one or two α-1,6-linked mannose residues and a few short side chains of α-1,2-linked mannose residues constitute the mannan in C. krusei (70, 73, 74). While C. parapsilosis mannan consists of both α-1,2- and α-1,3-linked mannose residues such as are found in C. albicans, it is not comprised of 1-*O*-α-phosphorylated units (75). In addition, contrasting with the other pathogenic *Candida* species, C. auris mannan is predominantly comprised of β-1,2-linked mannose residues (76). These differences in the structure and composition of mannan are likely to underscore the variations in the growth activity that were observed when different *Candida* species were incubated with Q-GRFT. Overall, these findings suggest that Q-GRFT’s anti-*Candida* activity may be beneficial as an additional strategy or alternative to the current antifungal treatment, given that growth-inhibitory activity was observed in these different strains despite the structural differences in their cell wall composition, as well as their documented innate resistance to common antifungal agents.

Mucosal transmission of HIV and viral shedding in genital secretions is increased in coinfection with pathogens causing sexually transmitted infections (STIs), including Neisseria gonorrhea and Chlamydia trachomatis (77, 78). Importantly, both these pathogens express mannosylated glycoproteins in their outer membranes (79, 80) that can act as ligands for lectins (81). The Neisseria gonorrhea cell wall envelope is rich in β-linked *N*-acetyl-d-glucosamine (d-GlcNAc) and β-d-galactosyl (β-d-Gal), which promote interaction with wheat germ agglutinin and ricin lectins. Additional structural polysaccharide components α-*N*-acetyl-d-galactosamine (α-d-GalNAc) and β-d-Gal linked to GalNAc and/or GlcNAc promote interaction with soybean agglutinin, peanut agglutinin, and Dolichos biflorus lectins (82). Chlamydia trachomatis possesses terminal mannose structures on the surface and in elementary bodies (EBs). These structures interact with and bind the lectin from Galanthus nivalis (GNA), with a resultant GNA-dependent inhibition in the number of intracellular inclusions (EBs). The GNA lectin prevented Chlamydia trachomatis infection of McCoy cells (83). In our study, we did not demonstrate any inhibitory effect of Q-GRFT on Neisseria gonorrhea and Chlamydia trachomatis. However, unlike the GNA lectin (83), Q-GRFT was neither toxic nor inhibitory to the growth of McCoy cells. Our findings do not demonstrate any inhibitory impact of Q-GRFT on the growth of Neisseria gonorrhea and Chlamydia trachomatis.

In conclusion, we report here the first demonstration of Q-GRFT’s antifungal activity, with growth inhibition of both C. albicans and non-albicans Candida species, including some multi- and pandrug-resistant strains of C. auris. Q-GRFT does not impact the growth of resident gut bacterial microbiome components. We have identified that Q-GRFT binds to α-mannan, with a resultant induction of reactive oxidative species that damage cellular structures. These findings underscore the need to further explore this lectin’s antifungal mechanism *in vivo* and support future development of Q-GRFT as an antifungal agent.

## MATERIALS AND METHODS

### Griffithsin-M78Q expression and product formulation.

The active pharmaceutical ingredient (API) used in the experiments was recombinantly expressed in the Nicotiana benthamiana plant-based system, as described previously (84). Because the original plant-produced recombinant Griffithsin protein is prone to oxidation at methionine-78, a single amino acid substitution replacing the met-78 residue in the nonbinding domain with glutamine (Q) has been performed, generating a stable variant, Q-GRFT (unpublished data). The M78Q variant is more stable against oxidation than the parent molecule and retains the potency and other characteristics of the parent protein (unpublished data). The API was dissolved in PBS solution, pH 7.4 at 10 ± 2 mg/ml, and diluted as needed for use in these experiments.

### Bacterial growth inhibition assays.

Gut and rectal microbiome and mycobiome components were incubated in broth medium in the presence of various concentrations of Q-GRFT for 24 to 72 h to determine the lectin’s growth-inhibitory effects. The microbiome components were as follows: Escherichia coli K-12 was incubated in Miller’s Luria broth (LB broth), while Lactobacillus acidophilus and Lactobacillus casei were incubated in De Man, Rogosa, Sharpe (MRS) broth. Bacteroides fragilis strains (nontoxigenic Bacteroides fragilis 9343 and enterotoxigenic Bacteroides fragilis 086) were incubated in brain heart infusion medium supplemented with clindamycin (BHI-clindamycin). Clostridium difficile ATCC 51695 and Bifidobacterium longum subsp. *longum* ATCC 15707 were incubated in brain heart infusion-supplemented (BHIS) medium and MRS–l-cysteine-enriched medium, respectively. Bacterial growth was measured as the optical density at 600 nm (OD_600_), monitored using a Synergy HT BioTek plate reader.

### MIC and cytotoxicity determination for Q-GRFT against Chlamydia and *Neisseria gonorrhea.*

For determination of Q-GRFT’s growth-inhibitory activity against Chlamydia trachomatis, 100 μl of McCoy cells (density of 5 × 10^4^ cells/well) was plated in a 96-well plate in Eagle’s minimum essential medium (EMEM) with 10% fetal bovine serum (FBS) and glutamine for 24 h. Chlamydia trachomatis was then added, and the culture centrifuged to promote infection. Doxycycline or Q-GRFT was added to the supernatant in triplicate. Cells were then fixed and stained with iodine in absolute methanol and glycerol. The wells were then observed for the presence of inclusion bodies. To establish Q-GRFT’s cytotoxicity to McCoy cells, the McCoy cell density was determined upon incubation with Q-GRFT.

### *Candida* growth inhibition assays.

To determine fungal inhibition, 1.0 × 10^5^ cells/ml of Candida albicans ATCC 32032, Candida glabrata CDC316, Candida krusei CDC397, Candida parapsilosis CDC337, Candida auris CDC383, Candida auris CDC384, Candida auris CDC385, Candida auris CDC386, Candida auris CDC388, and Candida auris CDC389 were incubated with various concentrations of Q-GRFT at 37°C in Sabouraud dextrose culture medium. Fungal growth was determined periodically up to 72 h using either a Bio-Rad TC10 automated cell counter (Bio-Rad Laboratories, Singapore) or an ECHO Rebel hybrid microscope (RBLTEW31; San Diego, CA, USA).

### Fluorescence microscopy.

Candida albicans (1.0 × 10^5^ cells/ml) was incubated with fluorescein-labeled 7.8 μM Q-GRFT overnight, followed by DAPI (4′,6-diamidino-2-phenylindole) staining. The cells were then fixed in 37% formaldehyde, mounted on glass slides, and visualized using an AxioScope.A1 microscope (Carl Zeiss MicroImaging GmbH).

### Fluorescence-based binding assay.

C. albicans (1.0 × 10^5^ cells/ml) was incubated with fluorescein-labeled 7.8 μM Q-GRFT overnight and washed in PBS, and the fluorescence intensities at excitation/emission wavelengths of 480/520 nm determined using a BioTek Synergy HT plate reader.

### Detection of hydrogen peroxide and ROS.

The staining procedure using DAB (3,3′-diaminobenzidine) (47) was employed to detect hydrogen peroxide in Candida albicans cells following incubation with Q-GRFT. Briefly, C. albicans (1.0 × 10^5^ cells/ml) was incubated for 2 h on microslides with 7.8 μM Q-GRFT, PBS (negative control), or 300 mM hydrogen peroxide (positive control) in the presence of 0.5 mg/ml DAB. The formation of hydrogen peroxide within cells, as brown pellets, was evaluated microscopically. A count to determine the percentage of brown-stained cells following Q-GRFT treatment in comparison with the control was performed from at least 3 independent experiments in triplicate.

The direct fluorescence method using H_2_DCF-DA (48) was used to assess the induction of ROS in C. albicans cells following incubation with Q-GRFT. Briefly, C. albicans was grown overnight from stock solution to achieve growth in the exponential phase, followed by inoculation into a fresh peptone-dextrose medium. Cells were then incubated with Q-GRFT (7.8 μM or 0.78 μM) overnight. Cultures were harvested, and cell numbers optimized and kept constant for all treatment groups. Harvested cells were then centrifuged at 5,000 to 6,000 × *g* for 10 min, followed by washing the pellet twice in PBS. Cells were then incubated for 30 min with H_2_DCF-DA (10 μM) in PBS in the dark, followed by centrifugation. The pellet was washed 2 times and resuspended in 200 μl PBS in a black 96-well plate. Fluorescence intensity was determined with excitation and emission wavelengths at 504 nm and 524 nm, respectively, using a Synergy HT BioTek plate reader.

### Ability of Q-GRFT to bind to gp120 and cell wall components.

Q-GFT’s ability to bind to gp120 and C. albicans cell wall components was determined using an ELISA. Briefly, a 96-well MaxiSorp Nunc plate was coated overnight with 0.1 ml per well of gp120, chitin, α-mannan, or β-glucan. The plate was then blocked for 2 h using 3% bovine serum albumin (BSA) in 1× PBS-Tween 20 (PBS-T) at 0.3 ml per well. Q-GRFT was then added at a concentration of 250 ng/ml, with 2- and 3-fold dilutions performed down the plate. The plate was incubated for 1 to 2 h at room temperature. A primary antibody, rabbit anti-GRFT antibody (0.1 ml per well, diluted 1:10,000 in 1× PBS), was added and the plate incubated for 1 h. A secondary antibody, goat anti-rabbit antibody (0.1 ml per well, diluted at 1:25,000 in 1× PBS) (4030-05; Southern Biotech) was added, followed by incubation for 1 h. TMB (3,3′,5,5′-tetramethylbenzidine) solution (0.1 ml per well) was then added for 4 min, and the reaction stopped with 0.1 ml per well of 1 N sulfuric acid. Absorbance was read at 450 nm using the Synergy HT BioTek plate reader.

### Viability assays.

To determine the impact of Q-GRFT on C. albicans viability, the trypan blue exclusion test of cell viability (85) was used. Briefly, C. albicans was incubated with Q-GRFT (7.8 μM) at 37°C overnight, followed by staining with 0.4% trypan blue (1 part trypan blue and 1 part cell suspension). The mixture was allowed to incubate at room temperature for ∼3 min, followed by cell evaluation by optical microscopy.

### Flow cytometry.

C. albicans viability analysis was performed using flow cytometry with BD Horizon fixable viability stain 780 (FVS780). Following overnight incubation with either Q-GRFT or PBS control, cells were added to complete RPMI medium (supplemented with 1 M HEPES, penicillin/streptomycin, fetal bovine serum, and 2-mercaptoethanol), filtered, and centrifuged for 5 min at 1,600 rpm. One to two million cells were then added to appropriate flow cytometry tubes, followed by washing with fluorescence-activated cell sorting (FACS) buffer (BD Biosciences, San Diego, CA) for 5 min at 1,600 rpm. Cells were then blocked with 2 μl of CD16/32 antibody (BioLegend, San Diego, California) for 10 min at 4°C, followed by staining with the viability dye and incubation at 4°C for 30 min. Cells were then fixed, washed, resuspended in 300 μl of FACS buffer, and analyzed using a BD device (LSR Fortessa; BD, USA), following the manufacturer’s instructions. Data were analyzed using FlowJo software (Tree Star, Inc., Ashland, OR).

### MIC determination.

The MIC_50_s and MIC_90_s were determined by the broth dilution method as described by the European Committee on Antimicrobial Susceptibility Testing (EUCAST) (86).

### SEM.

C. albicans cells were grown overnight in the presence of either PBS or 7.8 μM Q-GRFT in Sabouraud dextrose medium at 37°C. The medium was then rinsed off and cells fixed for 24 h at 4°C with scanning electron microscopy (SEM) fixative (2.5% glutaraldehyde, 2.5% formaldehyde, 0.1 M sodium cacodylate, pH 7.4). Cells were then rinsed and serially dehydrated in ethanol. They were then critically dried using hexamethyldisilazane (HMDS). Cells were then coated with gold/palladium in a Cressington 108auto/SE sputter coater, followed by examination using the Apreo 2 electron microscope.

### Candida albicans RNA extraction and sequencing.

A total of 4.0 × 10^7^ yeast cells were used for RNA extraction using the Qiagen RNeasy midi kit, following the manufacturer’s instructions. DNase treatment was performed using the RNase-free DNase set purchased from Qiagen. RNA quantification was carried out spectrophotometrically at 260 nm and 280 nm using a NanoDrop 1000 spectrophotometer (Thermo Scientific, USA). For RNA sequencing, pairwise comparisons were made between treated groups (7.8 μM Q-GRFT [1QG], 0.78 μM [2QG], and 7.8 μM Q-GRFT^lec neg^ [3QG]) and vehicle control (4VC). Sequencing was performed as follows.

1. Library preparation. Libraries were prepared using the Illumina stranded mRNA prep, ligation (catalog number 20040532; Illumina), and IDT for Illumina RNA UD indexes set B, ligation (catalog number 20040554; Illumina).
  - Purify and fragment mRNA. mRNAs were purified from 200 ng of total RNA samples with oligo(dT) magnetic RNA purification beads and denatured for 5 min at 65°C. Then, the supernatant was discarded, and the beads were washed with bead wash buffer. Captured polyadenylated RNAs were eluted using elution buffer at 80°C for 2 min. mRNAs were further purified in a second bead cleanup and then fragmented and primed during elution by adding 19 μl of fragmentation mix to the beads and incubating for 8 min at 94°C. After fragmentation, 17 μl of supernatant was removed from the beads, and synthesization of first-strand cDNA proceeded immediately thereafter.
  - Synthesize first-strand cDNA. Following the protocol, 8 μl of first-strand synthesis Act D mix and reverse transcriptase were added to each sample and the mixture heated on a thermocycler using preprogrammed conditions to produce first-strand cDNA from the hexamer-primed RNA fragments.
  - Synthesize second-strand cDNA. Second-strand marking mix was added and mixed well, and the mixture incubated at 16°C for 1 h. Blunt-ended, double-stranded cDNA fragments were then purified using Agencourt AMPure XP beads at 1.8×. An amount of 17.5 μl of eluate was collected and stored at −20°C.
  - Adenylate 3′ ends. Purified samples were mixed with 12.5 μl A-tailing mix and then incubated on the preprogrammed thermal cycler. An adenine (A) nucleotide was added to the 3′ end of each blunt fragment.
  - Ligate anchors. Ligation mix and RNA index anchors were added, and the mixture incubated in a preheated thermocycler at 30°C for 10 min. Stop ligation buffer was immediately added to each sample and mixed well.
  - Clean up fragments. The ligated fragments were purified using Agencourt AMPure XP Beads at 0.8×. An amount of 20 μl of the eluate was collected and used for library amplification.
  - Amplify library. Twelve cycles of PCRs were performed to selectively amplify the anchor-ligated DNA fragments and to add indexes and primer sequences for cluster generation. Samples were barcoded with IDT for Illumina DNA/RNA UD indexes as listed in Table 2. A complete list of the barcode sequences can be obtained from the Illumina support site Illumina Adapter Sequences.
  - Clean up library. Amplified libraries were purified using Agencourt AMPure XP beads at 1.0×. Amounts of 15 μl of eluted libraries were collected and stored at −20°C.
  - Validate library. The concentrations of libraries were measured by using the Qubit double-stranded DNA (dsDNA) HS assay kit (catalog number Q32851; Invitrogen). Libraries were diluted and normalized to the optimal range for Agilent Bioanalyzer analysis using the DNA high-sensitivity kit (catalog number 5067-4626; Agilent Technologies).
  - Normalize and pool libraries. The same numbers of libraries were pooled based on the molar concentration from Bioanalyzer.
2. Library denaturing and diluting for MiSeq nano 300. Pooled library was run on MiSeq to test quantity and quality, using the MiSeq reagent nano kit version 2, 300 cycles (catalog number MS-103-1001; Illumina). Library and PhiX control (catalog number FC-110-3001; Illumina) were denatured and diluted, using the standard normalization method and following the manufacturer’s directions, to a final concentration of 12.5 pM. Amounts of 300 μl of library and 300 μl of PhiX were combined and sequenced on the Illumina MiSeq.
3. Library repool. Based on MiSeq results, equal amounts of libraries were repooled for NextSeq run.
4. Library denaturing and diluting for NextSeq 500. Library and PhiX were denatured and diluted using the standard normalization method following the manufacturer’s directions.
5. Sequencing run. Sequencing was performed at the University of Louisville Brown Cancer Center Genomics Core on the Illumina NextSeq 500 using the NextSeq 500/550 75-cycle high-output kit version 2.5 (product number 20024906). The total volume of each library was 1.3 ml at 1.8 pM, with 1% PhiX spike-in. Two runs were made for the analysis described in this manuscript.

**TABLE 2:** Sample and barcode information^*a*^

Sample ID	17 Index ID	Index 1	15 Index ID	Index 2
1QG1_Candida	UDP0121	AGAGAACCTA	UDP0121	GGTTATGCTA
1QG2_Candida	UDP0122	GATATTGTGT	UDP0122	ACCACACGGT
1QG3_Candida	UDP0123	CGTACAGGAA	UDP0123	TAGGTTCTCT
2QG1_Candida	UDP0124	CTGCGTTACC	UDP0124	TATGGCTCGA
2QG2_Candida	UDP0125	AGGCCGTGGA	UDP0125	CTCGTGCGTT
2QG3_Candida	UDP0126	AGGAGGTATC	UDP0126	CCAGTTGGCA
3QG1_Candida	UDP0127	GCTGACGTTG	UDP0127	TGTTCGCATT
3QG2_Candida	UDP0128	CTAATAACCG	UDP0128	AACCGCATCG
3QG3_Candida	UDP0129	TCTAGGCGCG	UDP0129	CGAAGGTTAA
4VC1_Candida	UDP0130	ATAGCCAAGA	UDP0130	AGTGCCACTG
4VC2_Candida	UDP0131	TTCGGTGTGA	UDP0131	GAACAAGTAT
4VC3_Candida	UDP0132	ATGTAACGTT	UDP0132	ACGATTGCTG

a Bioinformatics data analysis was performed using the pipeline shown in Fig. 9. ID, identification number.

**FIG 9:**
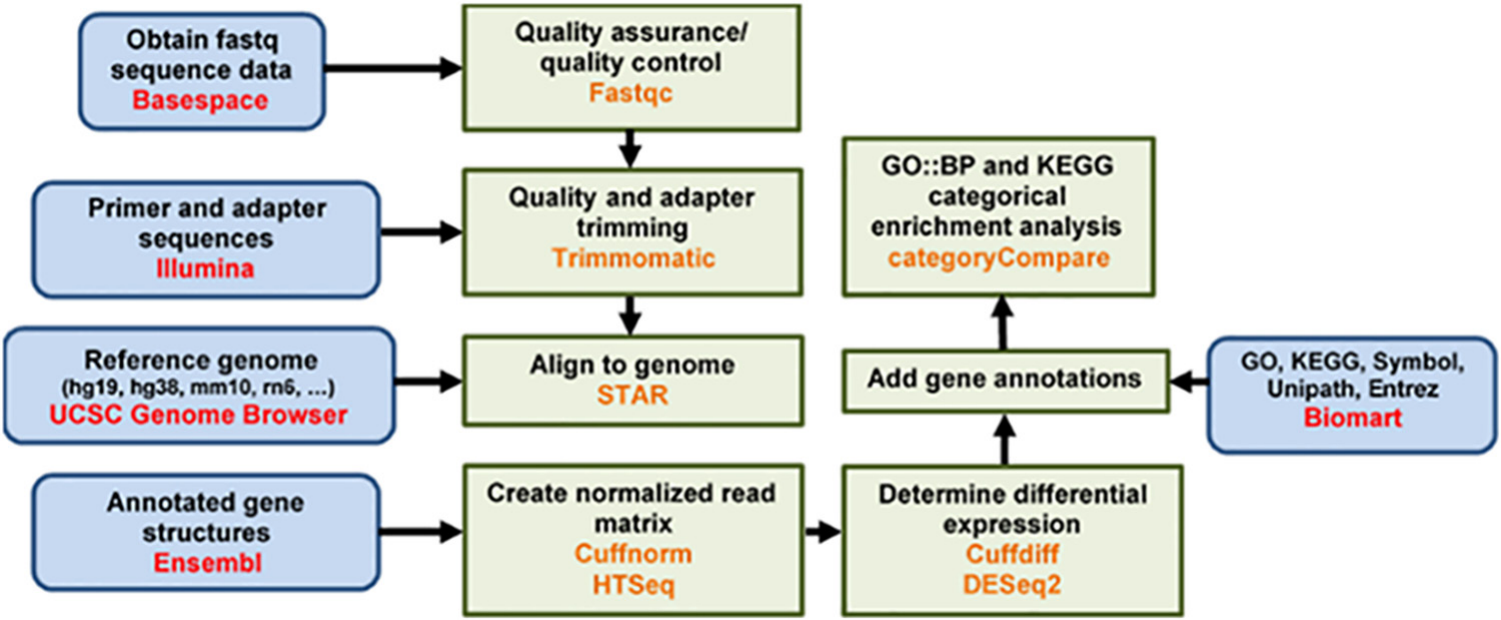
Bioinformatics analysis workflow diagram.

### Statistical analysis.

Statistical analyses were performed using GraphPad Prism version 7. Numerical data are presented as mean values ± standard deviations (SD) or mean values ± standard errors of the means (SEM). The number of repetitions and replicates of each assay and experiment are reported individually in the descriptions of each of the findings. Statistical differences between sets of data were evaluated using analysis of variance (ANOVA). *P* values of ≤0.05 were considered significant.
