# Supplementary material for: Novel Antifungal Activity of Q-Griffithsin, a Broad-Spectrum Antiviral Lectin
Source: Microbiol Spectr. 2021 Sep 8;9(2):e00957-21. doi: 10.1128/Spectrum.00957-21 (PMC8557872; doi:10.1128/Spectrum.00957-21)
Supplement: SUPPLEMENTAL FILE 1 — Supplemental material. Download SPECTRUM00957-21_Supp_1-8_seq1.pdf, PDF file, 0.6 MB [file spectrum00957-21_supp_1-8_seq1.pdf]

Supplementary figures and tables

**Compound (µg/mL)**	***Chlamydia trachomatis***	***Neisseria gonorrhea***
Q-GRFT	>50	>50
Doxycycline	0.03	-
Penicillin	-	0.03

Table S1 MIC results for Q-GRFT against *Chlamydia trachomatis* and *Neisseria gonorrhea*.

		1	0.5	0.25	0.125	0.0625	0.03	0.02	0.008	0.004	0.002	Pos Ctrl
**Doxycycline (µg/mL)**	**Cell Control**	-	-	-	-	-	-	-	-	-	-	-
	**Experimental**	-	-	-	-	-	-	+	+	+	+	+
		-	-	-	-	-	-	+	+	+	+	+
		-	-	-	-	-	-	+	+	+	+	+
**Griffithsin-M78Q (µg/mL)**		+	+	+	+	+	+	+	+	+	+	+
		+	+	+	+	+	+	+	+	+	+	+
	**Experimental**	+	+	+	+	+	+	+	+	+	+	+
	**Cell Control**	-	-	-	-	-	-	-	-	-	-	-
		50	25	12.5	6.25	3.125	1.563	0.781	0.391	0.195	0.098	Pos Ctrl

Table S2 MIC activity of Q-GRFT and doxycycline against *Neiserria gonorhoea*. There was no detectable impact of Q-GRFT on the growth of *Neiserria gonorhoea*.

Input number	P-Value	Corrected P-Value	Input/ Gene name
260	3.40E-15	2.95E-13	1|FDH3|FDH1|NDH51|SHM1|PAD1|PGI1|PSD1|OCH1|ERG27|PYC2|ERG25|AAH1|GLO1|GLO2|FAD3|SPT10|MET14|MET16|GRE3|ATP2|ALD5|RIB5|OPI3|UGA2|IDP1|GUK1|TKL1|ECM42|SPE1|URA7|URA5|PHHB|YCP4|TPI1|GPI8|ICL1|PDE1|FBP1|NIT3|ERG5|ERG6|ALG9|GNA1|ERG2|ERG3|GUT1|ALG6|ERG9|PSA2|ANP1|XOG1|FEN1|MET1|TPS2|LYS21|MIS12|MIS11|GPH1|GPM2|ECM38|FBA1|PMT1|CHS3|APT1|CHA1|SER33|LAB5|HEM2|HEM3|HEM1|SRB1|SOL3|OLE1|PGK1|ARO1|HEM13|CHS1|SER1|ARO9|DAO1|PGM2|PFK2|HIS4|HIS5|ZWF1|SOU2|GSY1|GLX3|HIS3|ACO1|ACS2|ARG8|MET3|NTH1|MET6|GTT11|ARG1|GTT12|CHO2|CDC21|CHT1|GLT1|GPX3|DAK2|CHT4|GSH2|COX11|PDX3|COX15|COX17|ERG4|AAT1|PUT1|GDH3|ECM17|NUC2|RHR2|PMI1|PMA1|FAA4|ALI1|AMO1|GAD1|ROT2|ERG1|RNR21|GAL1|CAR2|CAR1|MEU1|LRO1|GLC3|GLK4|ERG20|ATP1|GLK1|TRP3|HSX11|TRP1|GSC1|TRP5|PHO100|TAL1|LSC1|FAS2|GCV1|GCV2|GCV3|GAL102|CIS2|LYS9|THI20|CYR1|MVD|ALG11|LYS2|RNR1|QCR8|APA2|LYS12|ADH1|XKS1|GCY1|ADH5|ERG26|ERG251|HOM3|RBK1|HST3|LCB2|LYS22|DLD2|BNA4|HST1|MRF1|MET10|GWT1|FAD2|MCD4|CIT1|AIP2|GAL10|CYS4|SAM2|THI13|CYS3|SAM4|CYB2|TPS1|HIS1|GST2|GRE2|PFK26|VMA13|MES1|CHT3|COX4|MNN22|CHT2|ADE12|IDH2|YNK1|ARO10|RIB3|ADE6|MLS1|SGA1|GUA1|NCE103|GOR1|FAS1|ILV6|ILV1|GCA2|ILV3|RIB4|LEU4|LEU2|LEU1|BTS1|NPT1|GST3|GDB1|DUT1|ACC1|PRS1|GSL2|POT1-2|YSA1|GTT1|RIP1|ARD|ERG13|ERG10|KGD2|FAA21|ETR1|SAH1
78	5.57E-15	2.95E-13	RPL37B|RPS12|RPS21B|RPS10|RPS25B|RPL23A|RPS4A|RPL6|RPS9B|RPL40B|RPS18|RPL2|RPL3|RPL9B|RPL16A|RPL32|RPL24A|RPS3|RPL8B|RPS1|RPL27A|RPL14|MRP2|RPS14B|RPL42|RPS30|RPS21|RPL29|RPS15|RPL25|RPS19A|RPS17B|RPL82|RPS42|RPP2A|RPP2B|RPL20B|RPS6A|RPS27|RPS13|RPL5|RPS22A|RPL17B|RPS16A|RPS26A|RPS5|RPL43A|UBI3|RPL19A|MRPL6|RPP0|RPS23A|RPL11|RPL12|RPL13|RPS27A|RPL18|RPL28|YST1|RPL15A|RPS20|RPS28B|RPL38|RPL39|RLP24|RPL35|RPS24|RPL4B|RPL30|RPP1A|YML6|MRPL40|RPL10A|RPP1B|RPS7A|RPL10|RPL21A|RPS8A
115	1.38E-10	4.89E-09	PST3|PST2|PST1|LAT1|TKL1|SPE1|TPI1|FBP1|HXK2|PMM1|FUM11|MDH1-1|FDH3|SHM1|PAD1|PGI1|PSD1|ERG20|SPT10|IDH2|RIB3|RIB5|OPI3|IDP1|ECM42|YCP4|SMP2|ICL1|ERG6|ERG1|ERG3|GUT2|ERG9|PSA2|FEN1|GPH1|GOR1|FBA1|HEM2|HEM3|HEM1|SRB1|SOL3|PGK1|ARO1|ILV6|ARO9|PGM2|HIS4|HIS5|ZWF1|HIS1|HIS3|ACO1|ACS2|ARG8|MET1|MET6|ARG1|CHO2|GLT1|COX15|AAT1|PUT1|PMI1|AMO1|GAD1|CAR2|CAR1|GLC3|GLK4|GLK1|TRP3|TRP1|TRP5|PHO100|TAL1|LSC1|GCV1|GCV2|GCV3|LYS9|MVD|LYS2|ADH1|ADH5|HOM3|GPM2|CHA1|CIT1|GAL10|GPD2|SAM2|SAM4|YNK1|DPP3|ALD5|ADE6|MLS1|HEM13|ILV1|PFK2|ILV3|RIB4|LEU4|LEU2|LEU1|BTS1|GDB1|PRS1|POT1-2|ERG13|ERG10|KGD2|ACC1
91	7.66E-10	2.03E-08	LAT1|TKL1|SPE1|TPI1|FBP1|HXK2|PMM1|UGP1|FUM11|MDH1-1|FDH3|SHM1|PGI1|ERG20|ERG27|ERG26|ERG25|IDH2|ALD5|RAM2|IDP1|ECM42|ERG4|ERG5|ERG6|ERG1|ERG2|ERG3|ERG9|FBA1|SER33|SOL3|PGK1|ARO1|SER1|ARO9|DAO1|PGM2|HIS5|ZWF1|ACO1|ARG8|MET3|ARG1|GLT1|AAT1|PUT1|PMI1|CAR2|CAR1|GLK4|GLK1|TRP3|TRP1|TRP5|TAL1|LSC1|GCV1|GCV2|GCV3|GAL102|LYS9|MVD|LYS2|LYS12|ADH1|ADH5|ERG251|HOM3|GPM2|CHA1|CIT1|GAL10|CYS4|CYS3|LYS22|LYS21|YNK1|ADE6|ILV6|ILV1|PFK2|ILV3|ACS2|BTS1|PRS1|POT1-2|ERG13|ERG10|KGD2|ACC1
50	1.30E-07	2.76E-06	MCD1|SMC4|YCS4|CLB4|RAD53|CDC5|CDC6|CDC7|GRR1|CDC14|SMC1|SMC3|SMC2|DBF2|IRR1|CLN3|BFA1|PCL2|TUP1|CDC54|CDC55|GIN4|CEK2|BUB3|TEM1|YOX1|PCL1|CAK1|ORC3|ORC1|MCM6|BRN1|MCM3|MCM2|MPS1|SWE1|MOB1|DUN1|SSN6|GIN1|DDC1|CLB2|RFX1|CDC20|HSL1|DBF4|CDC45|SWI4|CDC47|CDC46
47	9.72E-06	0.000171773	AAT1|CIT1|HIS3|TKL1|TPI1|CYS4|SAM2|ECM42|CYS3|FBA1|GLT1|CAR1|LYS22|LYS21|SHM1|SER33|TRP3|TRP1|ARG8|PGK1|ARO1|TAL1|SER1|IDH2|ILV6|ILV1|PFK2|ILV3|HIS4|LYS9|LEU4|LEU2|HIS1|LEU1|ACO1|LYS2|PYC2|LYS12|MET6|HIS5|ARG1|PRS1|IDP1|HOM3|GPM2|CHA1|TRP5
42	5.32E-05	0.000805366	TPI1|AAT1|LAT1|CIT1|TKL1|MAE1|LSC1|FBP1|HXK2|FUM11|FBA1|FDH3|FDH1|SHM1|GLK4|PGI1|DAK2|GLK1|GCV1|SOL3|PGK1|MLS1|TAL1|SER1|IDH2|GCV2|GCV3|ILV1|PFK2|ACS2|ZWF1|KGD2|ACO1|PYC2|MDH1-1|PRS1|IDP1|GPM2|SER33|CHA1|ERG10|ICL1
21	0.000101	0.001297374	CDC46|POL3|RFA1|HYS2|POL2|RFC5|RNH35|RFC4|RFC3|RFC2|POL1|MCM6|DPB2|MCM2|POL32|MCM3|CDC47|PRI2|POL30|CDC54|RFA2
16	0.000122	0.001297374	RFC3|POL3|HYS2|RFA1|RFC5|RFC4|MSH2|EXO1|MLH3|MLH1|RFC2|MSH6|POL32|PMS1|POL30|RFA2
36	0.000122	0.001297374	HGT4|RAS1|EFH1|CLB4|TPK2|CDC5|CDC6|CDC7|CDC14|SMC1|RME1|SMC3|GLC7|IRR1|CLN3|RAD53|CDC54|UME7|ORC3|ORC1|MCM6|NDT80|MCM3|MCM2|SWE1|SNF1|DDC1|RTS1|CDC20|CYR1|DBF4|HGT6|CDC45|SWI4|CDC47|CDC46
18	0.0003	0.002652651	GPH1|TPS1|XOG1|SGA1|GDB1|GSC1|GSL2|PGM2|GLC3|GLK4|PGI1|TPS2|NTH1|GSY1|GLK1|HXK2|UGP1|GCA2
20	0.000318	0.002652651	GLK4|PMM1|GNA1|CHS3|CHS1|PGM2|MCR1|CHT1|PGI1|CHT3|GAL1|PSA2|GAL10|HXK2|GLK1|PMI1|SRB1|UGP1|CHT2|CHT4
15	0.000325	0.002652651	SOU1|FBA1|PFK26|PFK2|TPI1|PSA2|PMI1|GLK4|DAK2|SOU2|FBP1|HXK2|PMM1|SRB1|GLK1
33	0.00047	0.003555285	CST20|STT4|HST7|BMH1|YCK2|CLB2|GPD2|CPP1|GSC1|CAG1|STE50|HSL1|TUP1|BEM1|BEM3|ROM2|GRE2|SWE1|CLA4|MKK2|PKH3|SSU81|SSN6|BNR1|GSL2|SAC7|WSC2|STE4|PTC1|SWI4|CEK2|CPH1|CDC42
20	0.000546	0.003858921	CYB2|MLS1|LAT1|MDH1-1|GRE2|AIP2|ALD5|LYS22|GLX3|ACS2|MAE1|LEU4|LYS21|ERG10|GLO1|DLD2|ACC1|GLO2|PYC2|FUM11
12	0.001955	0.012949674	ERG4|ERG5|ERG6|ERG1|ERG2|ERG3|ERG9|ERG251|ARE2|ERG26|ERG25|ERG27
16	0.002732	0.017037083	SER1|GCV1|GCV2|GCV3|ILV1|DAO1|SHM1|GPM2|CHA1|SER33|HOM3|CYS4|AMO1|HEM1|CYS3|TRP5
16	0.003478	0.020483397	LYS12|ARG8|AAT1|IDH2|ILV6|IDP1|ILV3|HOM3|CIT1|LEU4|LEU2|LYS21|LEU1|ACO1|LYS22|ECM42
15	0.004604	0.02534002	RNR21|GST2|ECM38|GTT11|GTT12|SPE3|GPX3|CIS2|GTT1|ZWF1|IDP1|GSH2|RNR1|SPE1|GST3
18	0.004781	0.02534002	GLK4|ADH1|LAT1|FBA1|ADH5|FDH3|ALD5|PFK2|PGM2|ACS2|GPM2|PGI1|TPI1|FBP1|GAL10|HXK2|GLK1|PGK1
15	0.005803	0.029293732	SSB1|SSA2|SOD2|SOD3|SOD1|HST3|HST1|RPD31|SCH9|HSP104|CYR1|RAS1|HSP70|TPK2|PNC1
13	0.008103	0.039044024	GOR1|MLS1|MDH1-1|GCV1|GCV2|FDH1|SHM1|ERG10|CIT1|ACO1|ICL1|GCV3|ACS2
11	0.008645	0.039843302	FBA1|SER1|FDH3|FDH1|PFK2|SHM1|GPM2|DAK2|FBP1|ACS2|SER33
11	0.011238	0.049633697	TAL1|FBA1|PRS1|PFK2|PGM2|RBK1|PGI1|ZWF1|TKL1|FBP1|SOL3
10	0.01501	0.062631882	POL3|HYS2|RFA1|SGS1|RAD54|RAD52|RAD51|POL32|RDH54|RFA2
9	0.015363	0.062631882	GAL1|PFK2|PGM2|GLK4|GLK1|GAL10|HXK2|UGP1|GCA2
16	0.020586	0.080818073	AAT1|MET6|MDH1-1|SER1|MEU1|SPE3|SAH1|HOM3|SER33|CHA1|CDG1|SAM2|GSH2|CYS4|CYS3|SAM4
15	0.022785	0.084887309	RAD10|POL3|RAD23|RFA1|RAD14|RFC5|RFC4|RFC3|RFC2|DPB2|POL30|POL32|POL2|HYS2|RFA2
13	0.023224	0.084887309	FAD3|FAD2|OLE1|FAS2|ACC1|FAS1|POT1-2|FEN1|ERG10|FAA4|FAA21|ETR1|MRF1
9	0.025839	0.091297011	POL3|POL2|HYS2|APN2|DPB2|POL30|FPG1|POL32|OGG1
18	0.034592	0.114062106	RNR21|MET3|ADE6|MET14|YSA1|PRS1|PHO100|PGM2|APT1|ADE12|GUK1|PDE1|GUA1|YNK1|CYR1|RNR1|APA2|AAH1
22	0.036102	0.114062106	SES1|FRS1|FRS2|HTS1|NAM2|THS1|DED81|MES1|MST1|FMT1|MSK1|PRS|WRS1|VAS1|TYS1|MSW1|MSF1|GRS1|KRS1|ALA1|ILS1|CDC60
7	0.036586	0.114062106	ILV6|ILV1|ILV3|LEU4|CHA1|LEU2|LEU1
7	0.036586	0.114062106	IFE2|ILV6|UGA2|UGA1|ERG13|ERG10|GAD1
7	0.047248	0.143093162	FAS2|ACC1|FAS1|FAA4|FAA21|ETR1|MRF1
20	0.056411	0.166100009	VPS22|SSB1|VPS20|RVS167|VPS35|VPS27|SSA2|IST1|KIP4|VPS36|WAL1|CDC42|PEP8|ARC15|VRP1|HSP70|ARC40|ARC19|ARC18|VPS4
6	0.064524	0.184853389	LYS12|LYS22|LYS21|HOM3|LYS9|LYS2
8	0.066758	0.186219796	ADH1|AAT1|ADH5|ARO9|UGA2|HIS5|FDH3|AMO1
4	0.069186	0.188042793	CIS2|CDG1|GAD1|ECM38
10	0.075241	0.199388383	LAT1|MDH1-1|LSC1|IDH2|IDP1|KGD2|CIT1|ACO1|PYC2|FUM11
7	0.090091	0.232919454	MET1|HEM13|HEM2|HEM3|CYT2|HEM1|COX15
9	0.099047	0.248528043	ADH1|ADH5|FDH3|ALD5|POT1-2|ECI1|ERG10|FAA4|FAA21
6	0.100818	0.248528043	GCV1|CDC21|SHM1|FMT1|MIS12|MIS11
21	0.117476	0.278814798	MDN1|RIA1|UTP4|SIK1|BMS1|IMP4|NOP1|MPP10|NOP5|NOG1|RCL1|POP3|NOP10|GSP1|NHP2|UTP15|NMD3|CKB2|UTP18|RIX7|GAR1
6	0.122246	0.278814798	MET10|MET3|MET14|MET16|ECM17|APA2
4	0.124338	0.278814798	CST20|DBF2|MOB1|YCK2
4	0.124338	0.278814798	XKS1|ARD|UGP1|GRE3
7	0.127677	0.278814798	ARO1|AAT1|ARO9|HIS5|TRP3|TRP1|TRP5
9	0.131516	0.278814798	NIT3|AAT1|GDH3|GLT1|ARG1|UGA2|UGA1|ADE12|GAD1
9	0.131516	0.278814798	SPT10|AAT1|PUT1|CAR2|ALD5|CAR1|SPE3|DAO1|SPE1
5	0.138556	0.282441597	YCP4|PAD1|PST1|PST3|PST2
5	0.138556	0.282441597	ATM1|MLT1|HST6|CDR11|CDR4
6	0.145701	0.29140298	ARG8|AAT1|GDH3|ARG1|CAR1|ECM42
4	0.157094	0.302763895	MET3|MES1|MET6|CYS3
4	0.157094	0.302763895	MNT1|MNN2|MNT2|PMT1
5	0.16703	0.314007832	HIS4|HIS5|ALD5|HIS1|HIS3
9	0.168853	0.314007832	RPC40|RPB7|RPC11|RPA34|RPA135|RPC10|RPC31|RPC19|RPO26
8	0.193011	0.352743516	GCY1|GUT1|ALD5|LRO1|RHR2|DAK2|SMP2|DPP3
5	0.197559	0.35493699	FAD3|FAD2|FEN1|POT1-2|OLE1
12	0.211165	0.37305783	AYR1|AYR2|GUT2|PLB1|OPI3|CHO2|PSD1|PLB5|SMP2|GDE1|GPD2|DPP3
20	0.215788	0.374976385	TIF4631|MSN5|NMD3|MLP1|TIF5|SUB2|TIF3|SEC13|FUN12|RPG1A|GCD11|POP3|TEF2|GSP1|NAM7|TIF35|TIF34|TIF|HCR1|TIF11
6	0.226405	0.380616791	GRE2|LSC1|UGA1|ACS2|ERG10|ACC1
6	0.226405	0.380616791	RAM2|ERG20|MVD|ERG13|ERG10|BTS1
5	0.229806	0.380616791	LYS9|ALD5|KGD2|UGA2|ERG10
6	0.256024	0.417515364	PHO100|HST3|HST1|UGA2|NPT1|PNC1
5	0.263419	0.418075214	ARO9|AMO1|AAT1|ARO10|HIS5
3	0.272143	0.418075214	GLT1|NCE103|GDH3
3	0.272143	0.418075214	CIS2|ECM38|SHM1
3	0.272143	0.418075214	FEN1|ETR1|MRF1
8	0.291258	0.441047732	RNR21|URA7|PHO100|URA5|DUT1|CDC21|YNK1|RNR1
3	0.321412	0.479854238	CEK2|RAS1|CDC42
5	0.333327	0.490731024	ALD5|ERG13|UGA1|ERG10|POT1-2
4	0.350703	0.50924028	HSX11|LCB2|ISC1|YDC1
2	0.394371	0.564910251	YKU80|CAS1
3	0.41928	0.592582816	RIB3|RIB5|RIB4
5	0.439945	0.613607767	AMO1|ALD5|GAD1|UGA1|SPE3
2	0.456359	0.628234488	AYR1|AYR2
10	0.50525	0.673295309	SOD2|SOD3|SOD1|DAO1|POT1-2|ECI1|IDP1|FAA4|FAA21|PEX12
4	0.510212	0.673295309	IMP2|KAR2|SEC61|SEC62
3	0.511978	0.673295309	THI20|THI13|THI4
2	0.514499	0.673295309	AHP2|AHP1
4	0.547451	0.707680866	ALD5|BNA4|KGD2|ERG10
7	0.56225	0.717211298	RPN1|RPN2|RPN7|PUP1|RPT1|PRE8|RPN10
2	0.568356	0.717211298	PDX3|SER1
15	0.578054	0.720867951	YDJ1|KAR2|HSP90|SSA2|RAD23|SSB1|MSI3|SEC13|ROT2|SEC61|SEC62|EMP46|CDC48|PGA63|HSP70
3	0.634573	0.782147918	ILV6|LYS5|ILV3
2	0.662661	0.791398742	ABZ1|PHHB
5	0.662702	0.791398742	MNN22|ANP1|OCH1|ALG9|ALG11
1	0.664476	0.791398742	PMT1
3	0.70344	0.828496304	SEC20|VAM3|VTI1
1	0.720336	0.834562724	GPX3
13	0.724337	0.834562724	VPS33|TPK2|UME7|PRB1|SCH9|AUT7|LCB2|VTI1|RAS1|GCN4|VAM3|SNF1|ARP3
1	0.766903	0.865088826	POT1-2
7	0.767154	0.865088826	SUB2|RTS1|GLC7|NAM7|CDC55|SWD2|SUP35
3	0.787464	0.869491012	MCD4|GWT1|GPI8
3	0.787464	0.869491012	STT4|TPI1|INP51
4	0.822967	0.899324568	ALG11|ALG9|ALG6|ROT2
5	0.835412	0.903609321	VMA13|VPS27|SEC61|TUB1|TUB2
5	0.850798	0.910954931	UME7|AUT7|CKB2|MKK2|WSC2
14	0.887963	0.941241307	ATP2|ATP1|NUC2|NDH51|COX4|VMA13|ATP20|PMA1|RIP1|COX11|QCR8|ALI1|COX15|COX17
2	0.90189	0.946537937	STT4|INP51
5	0.935612	0.97230229	GRR1|UBC8|UBC15|CDC34|CDC20
5	0.977293	0.995562087	NOT5|DHH1|CCR4|PFK2|EDC3
1	0.984877	0.995562087	AUT7
2	0.988436	0.995562087	NPL3|TBP1
6	0.995562	0.995562087	SSB1|SUB2|PRP22|SSA2|DBP2|HSP70

Table S3 KEGG pathway analysis of upregulated differentially expressed genes associated with treatment with 7.8 µM Q-GRFT (1QG).

Gene name/ Description	Log2FC	P-value	Adjusted P-value
RNR1, ribonucleotide-diphosphate reductase subunit	-2.145	3.14E-62	1.95E-58
hypothetical protein CAALFM_C102370CA	-2.255	2.07E-61	6.44E-58
PHO84, phosphate transporter	-1.907	2.76E-40	5.72E-37
RBR1, Repressed by RIM101 protein 1	-2.065	7.04E-38	8.76E-35
CAALFM_C107490CA, DNA-directed DNA polymerase alpha subunit	-1.516	2.01E-35	1.93E-32
CDC46, MCM DNA helicase complex subunit	-1.668	2.85E-35	2.22E-32
CTF18, Ctf18p	-1.7	4.92E-33	2.55E-30
POL1, DNA-directed DNA polymerase alpha catalytic subunit	-1.655	1.02E-32	4.87E-30
DUT1, bifunctional dITP/dUTP diphosphatase	-1.481	5.12E-32	2.27E-29
TRY6, Transcriptional regulator of yeast form adherence 6	-1.852	6.81E-31	2.82E-28
YBL053, Topoisomerase 1-associated factor 1	-1.382	9.75E-31	3.79E-28
TOS4, Tos4p	-1.308	8.96E-30	3.28E-27
hypothetical protein CAALFM_C201420CA	-1.432	9.69E-30	3.35E-27
SHA3, putative serine/threonine protein kinase	-1.055	2.79E-29	9.15E-27
CDC54, MCM DNA helicase complex subunit	-1.321	3.78E-29	1.18E-26
PGA45, Predicted GPI-anchored protein 45	-1.505	1.91E-28	5.41E-26
HCM1, Hcm1p	-1.598	1.22E-27	3.17E-25
SMC3, cohesin subunit	-1.79	1.59E-26	3.96E-24
hypothetical protein CAALFM_C501070CA	-1.306	8.46E-26	2.02E-23
CSM3, Chromosome segregation in meiosis protein 3	-1.108	4.48E-25	9.61E-23

Table S4. The top 20 down-regulated DEGs for 7.8 µM Q-GRFT treated *C. albicans* (1QG) vs non-treated control (VC) cells.

<table>
<tr><th>Term/ gene function</th><th>Input number</th><th>P-Value</th><th>Corrected P-Value</th><th>Input/ Gene name</th></tr>
<tr><td>Glycolysis / Gluconeogenesis</td><td>18</td><td>8.04E-11</td><td>6.59E-09</td><td>GLK4|ADH1|ADH5|FBA1|PGI1|FDH3|CDC19|FBP1|PGM2|GPM2|ENO1|TPI1|GPM1|GLK1|GAL10|TDH3|PDC11|PGK1</td></tr>
<tr><td>Biosynthesis of antibiotics</td><td>37</td><td>1.80E-10</td><td>7.38E-09</td><td>ERG4|GLY1|TKL1|TPI1|FBP1|GAL10|TDH3|UGP1|FUM11|PDC11|FBA1|FDH3|CDC19|GLK4|PGI1|CHA1|GLK1|AAT22|SOL3|PGK1|ADE6|TAL1|ILV5|ARO9|GND1|GAL102|PGM2|ENO1|ECM42|ACO2|INO1|ADH1|MDH1-1|ADH5|LYS2|GPM2|GPM1</td></tr>
<tr><td>Carbon metabolism</td><td>25</td><td>4.26E-10</td><td>9.23E-09</td><td>FDH1|TKL1|TPI1|FBP1|TDH3|FUM11|MDH1-1|FDH3|CDC19|GLK4|PGI1|DAK2|CHA1|GLK1|AAT22|SOL3|PGK1|MLS1|TAL1|ALD6|GND1|ENO1|FBA1|GPM2|GPM1</td></tr>
<tr><td>Biosynthesis of secondary metabolites</td><td>43</td><td>4.50E-10</td><td>9.23E-09</td><td>PST3|PST1|GLY1|TDH3|PSA2|TPI1|FBP1|GAL10|GPD2|GAD1|FUM11|PAD1|FBA1|FDH3|CDC19|GLK4|PGI1|CHA1|GLK1|AAT22|SOL3|PGK1|ADE6|MLS1|TAL1|ILV5|ARO9|GND1|HEM13|RIB5|PGM2|HIS4|ENO1|LEU4|TKL1|ECM42|ADH1|MDH1-1|ADH5|LYS2|GPM2|GPM1|PDC11</td></tr>
<tr><td>Metabolic pathways</td><td>74</td><td>3.28E-08</td><td>5.38E-07</td><td>PST3|ERG4|PST1|GLY1|LAG1|PSA2|THI4|TPI1|FBP1|GAL10|TDH3|GAD1|PNC1|FUM11|PAD1|AYR2|RNR1|FBA1|FDH3|CDC19|DES1|UGP1|GLK4|PGI1|DAK2|CHA1|GLK1|ALD6|GLO1|ARO10|AAT22|GND1|PGK1|FAD3|ADE6|MLS1|TAL1|CHS1|GRE3|ILV5|ARO9|TKL1|HEM13|RIB5|GAL102|PGM2|UGA2|HIS4|ENO1|LEU4|GSY1|GLX3|CHT2|ECM42|ACO2|INO1|MUQ1|ADH1|GCY1|MDH1-1|PHHB|DUT1|ADH5|LYS2|SOL3|GPM2|FDH1|GPM1|SUR2|GSH2|GLN1|ETR1|PDC11|MRF1</td></tr>
<tr><td>Biosynthesis of amino acids</td><td>20</td><td>1.73E-06</td><td>2.36E-05</td><td>HIS4|AAT22|TAL1|FBA1|ILV5|CDC19|LYS2|LEU4|TKL1|GPM2|ENO1|TPI1|GPM1|CHA1|TDH3|ECM42|ACO2|GLY1|GLN1|PGK1</td></tr>
<tr><td>Cell cycle - yeast</td><td>18</td><td>6.21E-06</td><td>7.28E-05</td><td>CDC46|BUB3|SKP1|SMC1|PCL2|SMC3|MCD1|IRR1|CDC20|CLB2|HSL1|CDC6|CDC45|SWI4|CDC54|GIN1|GIN4|MCM2</td></tr>
<tr><td>Methane metabolism</td><td>8</td><td>3.31E-05</td><td>0.000339349</td><td>FBA1|FDH3|FDH1|GPM2|ENO1|DAK2|GPM1|FBP1</td></tr>
<tr><td>Pentose phosphate pathway</td><td>8</td><td>4.38E-05</td><td>0.000399469</td><td>TAL1|FBA1|GND1|TKL1|PGI1|PGM2|SOL3|FBP1</td></tr>
<tr><td>Amino sugar and nucleotide sugar metabolism</td><td>9</td><td>0.000184872</td><td>0.001515947</td><td>CHS1|PGM2|GLK4|PGI1|CHT2|PSA2|GAL10|UGP1|GLK1</td></tr>
<tr><td>Fructose and mannose metabolism</td><td>7</td><td>0.000262244</td><td>0.001954909</td><td>FBA1|DAK2|PSA2|GLK4|TPI1|GLK1|FBP1</td></tr>
<tr><td>DNA replication</td><td>8</td><td>0.000700848</td><td>0.00478913</td><td>CDC46|POL3|RFA1|POL1|MCM2|CDC54|PRI2|POL30</td></tr>
<tr><td>Tyrosine metabolism</td><td>6</td><td>0.000923285</td><td>0.005823797</td><td>ADH1|ADH5|ARO9|UGA2|FDH3|AAT22</td></tr>
<tr><td>Mismatch repair</td><td>6</td><td>0.001423619</td><td>0.008338339</td><td>POL3|RFA1|MSH3|MLH3|MSH6|POL30</td></tr>
<tr><td>Galactose metabolism</td><td>5</td><td>0.002594287</td><td>0.0141821</td><td>GLK4|GLK1|PGM2|GAL10|UGP1</td></tr>
<tr><td>Pyruvate metabolism</td><td>7</td><td>0.004406274</td><td>0.021759599</td><td>MLS1|MDH1-1|CDC19|GLX3|LEU4|GLO1|FUM11</td></tr>
<tr><td>Meiosis - yeast</td><td>11</td><td>0.004511136</td><td>0.021759599</td><td>SMC1|SMC3|IRR1|CDC20|NDT80|MCM2|CDC45|SWI4|CDC54|CDC46|CDC6</td></tr>
<tr><td>Starch and sucrose metabolism</td><td>6</td><td>0.005662283</td><td>0.025794844</td><td>PGM2|GLK4|PGI1|GSY1|GLK1|UGP1</td></tr>
<tr><td>2-Oxocarboxylic acid metabolism</td><td>5</td><td>0.029849264</td><td>0.12882314</td><td>ILV5|AAT22|ECM42|ACO2|LEU4</td></tr>
<tr><td>Ubiquinone and other terpenoid-quinone biosynthesis</td><td>3</td><td>0.031978185</td><td>0.131110557</td><td>PST3|PAD1|PST1</td></tr>
<tr><td>Valine, leucine and isoleucine biosynthesis</td><td>3</td><td>0.038002666</td><td>0.148391361</td><td>ILV5|LEU4|CHA1</td></tr>
<tr><td>Sphingolipid metabolism</td><td>3</td><td>0.044561498</td><td>0.166092856</td><td>SUR2|LAG1|DES1</td></tr>
<tr><td>Fatty acid degradation</td><td>4</td><td>0.047190814</td><td>0.168245512</td><td>FDH3|ECI1|ADH1|ADH5</td></tr>
<tr><td>Glyoxylate and dicarboxylate metabolism</td><td>4</td><td>0.052483763</td><td>0.173462296</td><td>FDH1|GLN1|MLS1|MDH1-1</td></tr>
<tr><td>MAPK signaling pathway - yeast</td><td>8</td><td>0.054925956</td><td>0.173462296</td><td>CST20|BNR1|HST7|SAC7|CLB2|HSL1|GPD2|SWI4</td></tr>
<tr><td>Alanine, aspartate and glutamate metabolism</td><td>4</td><td>0.058086418</td><td>0.173462296</td><td>GLN1|AAT22|GAD1|UGA2</td></tr>
<tr><td>Phenylalanine metabolism</td><td>3</td><td>0.059231028</td><td>0.173462296</td><td>ARO9|ARO10|AAT22</td></tr>
<tr><td>Arginine biosynthesis</td><td>3</td><td>0.059231028</td><td>0.173462296</td><td>ECM42|AAT22|GLN1</td></tr>
<tr><td>Base excision repair</td><td>3</td><td>0.075861349</td><td>0.203317929</td><td>POL3|POL30|FPG1</td></tr>
<tr><td>Fatty acid elongation</td><td>2</td><td>0.079343582</td><td>0.203317929</td><td>ETR1|MRF1</td></tr>
<tr><td>Pentose and glucuronate interconversions</td><td>2</td><td>0.079343582</td><td>0.203317929</td><td>UGP1|GRE3</td></tr>
<tr><td>Sulfur relay system</td><td>2</td><td>0.079343582</td><td>0.203317929</td><td>AHP2|AHP1</td></tr>
<tr><td>Glycine, serine and threonine metabolism</td><td>4</td><td>0.08351796</td><td>0.204672932</td><td>GPM2|GPM1|GLY1|CHA1</td></tr>
<tr><td>Homologous recombination</td><td>3</td><td>0.084864387</td><td>0.204672932</td><td>POL3|RFA1|SGS1</td></tr>
<tr><td>Inositol phosphate metabolism</td><td>3</td><td>0.104139815</td><td>0.243984709</td><td>ALD6|TPI1|INO1</td></tr>
<tr><td>Nucleotide excision repair</td><td>4</td><td>0.138662484</td><td>0.314283434</td><td>POL3|POL30|RFA1|RAD14</td></tr>
<tr><td>Lysine biosynthesis</td><td>2</td><td>0.141810818</td><td>0.314283434</td><td>LYS2|ACO2</td></tr>
<tr><td>Butanoate metabolism</td><td>2</td><td>0.158708146</td><td>0.342475473</td><td>GAD1|UGA2</td></tr>
<tr><td>Cysteine and methionine metabolism</td><td>4</td><td>0.165831011</td><td>0.348670331</td><td>GSH2|AAT22|CHA1|MDH1-1</td></tr>
<tr><td>Fatty acid biosynthesis</td><td>2</td><td>0.175951786</td><td>0.360701161</td><td>ETR1|MRF1</td></tr>
<tr><td>Glutathione metabolism</td><td>3</td><td>0.207201876</td><td>0.414403753</td><td>GSH2|GND1|RNR1</td></tr>
<tr><td>Fatty acid metabolism</td><td>3</td><td>0.219829461</td><td>0.41920967</td><td>FAD3|ETR1|MRF1</td></tr>
<tr><td>Longevity regulating pathway - multiple species</td><td>3</td><td>0.219829461</td><td>0.41920967</td><td>SOD2|PNC1|SOD1</td></tr>
<tr><td>Peroxisome</td><td>4</td><td>0.235373384</td><td>0.438650397</td><td>TRP99|ECI1|SOD2|SOD1</td></tr>
<tr><td>Phenylalanine, tyrosine and tryptophan biosynthesis</td><td>2</td><td>0.265071765</td><td>0.48301966</td><td>ARO9|AAT22</td></tr>
<tr><td>Purine metabolism</td><td>4</td><td>0.277826805</td><td>0.493906069</td><td>ADE6|CDC19|RNR1|PGM2</td></tr>
<tr><td>Nicotinate and nicotinamide metabolism</td><td>2</td><td>0.283092503</td><td>0.493906069</td><td>UGA2|PNC1</td></tr>
<tr><td>Taurine and hypotaurine metabolism</td><td>1</td><td>0.290048826</td><td>0.495500077</td><td>GAD1</td></tr>
<tr><td>beta-Alanine metabolism</td><td>2</td><td>0.301072935</td><td>0.50383634</td><td>ALD6|GAD1</td></tr>
<tr><td>Ether lipid metabolism</td><td>1</td><td>0.323976279</td><td>0.531321097</td><td>AYR2</td></tr>
<tr><td>Nitrogen metabolism</td><td>1</td><td>0.356287389</td><td>0.561837806</td><td>GLN1</td></tr>
<tr><td>Hippo signaling pathway - multiple species</td><td>1</td><td>0.356287389</td><td>0.561837806</td><td>CST20</td></tr>
<tr><td>Other types of O-glycan biosynthesis</td><td>1</td><td>0.387058924</td><td>0.578629818</td><td>MNT2</td></tr>
<tr><td>Glycerolipid metabolism</td><td>2</td><td>0.389192623</td><td>0.578629818</td><td>DAK2|GCY1</td></tr>
<tr><td>Glycerophospholipid metabolism</td><td>3</td><td>0.390716796</td><td>0.578629818</td><td>MUQ1|AYR2|GPD2</td></tr>
<tr><td>Ubiquitin mediated proteolysis</td><td>3</td><td>0.403783587</td><td>0.578629818</td><td>UBC8|CDC20|SKP1</td></tr>
<tr><td>Citrate cycle (TCA cycle)</td><td>2</td><td>0.406269552</td><td>0.578629818</td><td>MDH1-1|FUM11</td></tr>
<tr><td>Endocytosis</td><td>4</td><td>0.409274749</td><td>0.578629818</td><td>VPS23|VRP1|AGE1|WAL1</td></tr>
<tr><td>Riboflavin metabolism</td><td>1</td><td>0.444272326</td><td>0.607172179</td><td>RIB5</td></tr>
<tr><td>Folate biosynthesis</td><td>1</td><td>0.444272326</td><td>0.607172179</td><td>PHHB</td></tr>
<tr><td>Pyrimidine metabolism</td><td>2</td><td>0.456039214</td><td>0.61303632</td><td>DUT1|RNR1</td></tr>
<tr><td>ABC transporters</td><td>1</td><td>0.470850232</td><td>0.622737404</td><td>MLT1</td></tr>
<tr><td>Histidine metabolism</td><td>1</td><td>0.496160956</td><td>0.635706225</td><td>HIS4</td></tr>
<tr><td>Thiamine metabolism</td><td>1</td><td>0.496160956</td><td>0.635706225</td><td>THI4</td></tr>
<tr><td>RNA degradation</td><td>3</td><td>0.5165475</td><td>0.646389511</td><td>DHH1|HSP60|ENO1</td></tr>
<tr><td>Biosynthesis of unsaturated fatty acids</td><td>1</td><td>0.520264729</td><td>0.646389511</td><td>FAD3</td></tr>
<tr><td>Lysine degradation</td><td>1</td><td>0.543218925</td><td>0.664835102</td><td>UGA2</td></tr>
<tr><td>Ribosome</td><td>6</td><td>0.554335672</td><td>0.668463604</td><td>RPP1A|RPP1B|RPL5|RPL3|RPL4B|RPP0</td></tr>
<tr><td>Pantothenate and CoA biosynthesis</td><td>1</td><td>0.565078202</td><td>0.671542211</td><td>ILV5</td></tr>
<tr><td>Porphyrin and chlorophyll metabolism</td><td>1</td><td>0.585894626</td><td>0.680395332</td><td>HEM13</td></tr>
<tr><td>Valine, leucine and isoleucine degradation</td><td>1</td><td>0.605717796</td><td>0.680395332</td><td>ALD6</td></tr>
<tr><td>Steroid biosynthesis</td><td>1</td><td>0.605717796</td><td>0.680395332</td><td>ERG4</td></tr>
<tr><td>Protein export</td><td>1</td><td>0.605717796</td><td>0.680395332</td><td>IMP1</td></tr>
<tr><td>Propanoate metabolism</td><td>1</td><td>0.624594958</td><td>0.692118737</td><td>ALD6</td></tr>
<tr><td>Arginine and proline metabolism</td><td>1</td><td>0.746532905</td><td>0.816209309</td><td>AAT22</td></tr>
<tr><td>Proteasome</td><td>1</td><td>0.828948876</td><td>0.894392209</td><td>RPN2</td></tr>
<tr><td>Aminoacyl-tRNA biosynthesis</td><td>2</td><td>0.854552311</td><td>0.910042721</td><td>HTS1|KRS1</td></tr>
<tr><td>Protein processing in endoplasmic reticulum</td><td>2</td><td>0.89736848</td><td>0.943387376</td><td>PGA63|SKP1</td></tr>
<tr><td>Spliceosome</td><td>1</td><td>0.976238653</td><td>0.981450678</td><td>PRP22</td></tr>
<tr><td>Autophagy - yeast</td><td>1</td><td>0.976238653</td><td>0.981450678</td><td>PRB1</td></tr>
<tr><td>Ribosome biogenesis in eukaryotes</td><td>1</td><td>0.977386419</td><td>0.981450678</td><td>NOP1</td></tr>
<tr><td>RNA transport</td><td>1</td><td>0.981450678</td><td>0.981450678</td><td>RPG1A</td></tr>
</table>

Table S5. KEGG pathway analysis of upregulated differentially expressed genes associated with treatment with 0.78 µM Q-GRFT (2QG).

Gene name/ Description	Log2FC	P-value	Adjusted P-value
hypothetical protein CAALFM_C201630WA	-0.941	6.53E-15	1.08E-11
MCD1, kleisin alpha	-1.165	6.93E-15	1.08E-11
hypothetical protein CAALFM_C102370CA	-0.907	7.20E-12	7.46E-09
CCN1, Ccn1p	-0.931	8.74E-11	5.44E-08
hypothetical protein CAALFM_C602090CA	-0.759	5.17E-10	2.48E-07
SHA3, putative serine/threonine protein kinase	-0.542	7.51E-09	2.46E-06
HMX1, Hmx1p	-0.984	8.32E-09	2.59E-06
PUT4, Put4p	-0.868	6.19E-08	1.43E-05
hypothetical protein CAALFM_C100090WA	-0.85	8.92E-08	1.91E-05
GIN4, protein kinase	-0.652	1.32E-07	2.64E-05
FRE7, Fre7p	-0.514	1.48E-07	2.88E-05
TNA1, Tna1p	-1.013	3.23E-07	5.43E-05
VID21, Vid21p	-0.804	4.63E-07	7.03E-05
RPP0, ribosomal protein P0	-0.751	5.68E-07	8.16E-05
CAS5, Cas5p	-0.386	5.77E-07	8.16E-05
hypothetical protein CAALFM_C503430WA	-0.698	6.09E-07	8.42E-05
RNR1, ribonucleotide-diphosphate reductase subun	-0.629	9.43E-07	0
MNT2, alpha-1	-0.379	1.38E-06	0
SMC3, cohesin subunit	-0.798	1.44E-06	0
AAT22, Aat22p	-0.608	1.73E-06	0

Table S6. The top 20 down-regulated DEGs for 0.78 µM Q-GRFT treated *C. albicans* (2QG) vs non-treated control (VC) cells.

Input number	P-Value	Corrected P-Value	Input/ Gene name
179	2.79E-12	2.88E-10	LAT1|IDI1|THI6|UGA1|THI4|AFP99|PLC2|CDG1|TDH3|PMM1|PNC1|AAH1|FCA1|ISC1|MDH1-1|FDH3|FDH1|NDH51|SHM1|PSD2|DAK2|ERG26|ERG25|ERG24|FAD3|FAD2|MET14|MET15|MET16|GPX2|IDH2|RIB5|UGA2|GUK1|SAH1|URA7|ADO1|URA3|IMH3|HYU1|TPS3|FBP1|NIT3|ERG5|ERG6|ERG1|ERG2|ERG3|GUT1|ALG1|ALG6|ERG9|PSA2|LCB2|FEN1|GPM2|ECM38|CPA2|CDC19|APT1|GPM1|SER33|LAB5|HEM1|SRB1|SOL3|OLE1|CHS1|SER1|PHO8|UAP1|MAL2|DAO1|PFK2|SOU2|GSY1|HIS3|MET1|ARG8|MET3|NTH1|MET6|GTT11|GTT12|ARG4|CDC21|CAR2|CHT3|TPS2|SLC1|CUP5|PUT1|GDH3|ECM17|NUC2|ALG8|RHR2|PMI1|FAA4|ALI1|GAD1|GNA1|RNR21|GAL1|FGR22|CAR1|MEU1|SDH1|XKS1|GCA2|PHO10
62	9.74E-08	5.02E-06	ERG5|ERG6|CAR1|LAT1|ERG1|ERG2|ERG3|SDH1|IDI1|SDH2|DQD1|ERG9|SER1|STE24|PMI1|FBP1|GAL10|TDH3|CYS4|PMM1|CYS3|MDH1-1|CAR2|FDH3|CDC19|SHM1|GPM1|SER33|ERG25|ERG24|SOL3|ERG26|ADE4|ARG8|ADE6|LSC2|MET15|LSC1|ILV5|IDH2|UAP1|GAL102|DAO1|POT1|PDC12|DAC1|ACS2|LYS12|MET3|PUT1|PRS1|ARG3|ARG4|POT1-2|ERG251|GPM2|CHA1|ERG13|ERG10|ERG11|ACC1|PFK2
37	3.66E-07	1.18E-05	LTE1|MCD1|YCS4|CDC5|CDC6|CDC7|GRR1|CDC14|SMC1|SMC3|SMC2|DBF2|IRR1|CLN3|BFA1|CDH1|DUN1|CDC55|GIN4|CEK2|BUB3|PCL1|MCM6|MCM3|MCM2|MPS1|APC1|CDC54|GIN1|CLB2|PPH21|HSL1|FAR1|CDC45|SWI4|CDC47|CDC46
21	4.57E-07	1.18E-05	CDC46|POL3|RFA1|HYS2|POL2|RFC5|RNH35|RFC4|RFC3|RFC2|POL1|MCM6|DPB2|MCM2|POL32|MCM3|CDC47|PRI2|POL30|CDC54|RFA2
70	4.96E-06	9.86E-05	ERG6|CAR1|LAT1|ERG1|PUT1|ERG3|SDH1|IDI1|GUT2|SDH2|DQD1|ERG9|PSA2|PMI1|GPD1|FBP1|GAL10|GPD2|SAM2|PMM1|GAD1|GOR1|MDH1-1|CAR2|FDH3|CDC19|PHO100|SHM1|PSD2|GPM1|ERG24|DPP1|HEM1|SRB1|SRT1|CDS1|ADE4|ADE6|LSC2|MET15|LSC1|ILV5|IDH2|RIB5|HEM15|PFK2|MET1|LEU2|POT1|HIS3|ACS2|SLC1|ARG8|DPP3|MET6|IMH3|PRS1|ARG3|ARG4|POT1-2|SOL3|GPM2|CHA1|ERG13|ERG10|ERG11|TDH3|FEN1|PDC12|ACC1
15	5.74E-06	9.86E-05	POL3|HYS2|RFA1|RFC5|RFC4|MSH2|EXO1|RFC3|RFC2|MSH6|MLH1|POL32|PMS1|POL30|RFA2
12	8.11E-05	0.001193733	ERG5|ERG6|ERG1|ERG2|ERG3|ERG24|ERG9|ERG251|ARE2|ERG26|ERG25|ERG11
27	0.000127646	0.001643436	GLC7|TOR1|RIM15|TPK2|CDC5|CDC6|CDC7|CDC14|SMC1|SMC3|HGT4|IRR1|CLN3|CDC54|UME7|MCM6|CYR1|MCM3|MCM2|APC1|PPH21|DLH1|NDT80|CDC45|SWI4|CDC47|CDC46
11	0.000549723	0.006291271	SER1|FDH3|FDH1|PFK2|SHM1|GPM2|DAK2|GPM1|SER33|ACS2|FBP1
15	0.000923345	0.009510457	RAD2|POL3|HYS2|RFA1|POL2|RFC5|RFC4|RFC3|RFC2|RFA2|DPB2|POL30|RAD1|POL32|RAD23
13	0.00101932	0.009544545	RNR21|ECM38|GCS1|GST1|GTT11|GTT12|CIS2|URE2|GPX3|GPX2|HYU1|RNR1|GST3
13	0.001292624	0.011095021	FAD3|FAD2|OLE1|FAS2|ACC1|FAS1|POT1-2|POT1|FEN1|ERG10|FAA4|ETR1|MRF1
26	0.002470464	0.019573677	FDH1|LAT1|SDH1|SDH2|SER1|FBP1|TDH3|MDH1-1|FDH3|CDC19|SHM1|DAK2|GPM1|SER33|IDH2|SOL3|LSC2|MET15|LSC1|ALD6|PFK2|ACS2|PRS1|GPM2|CHA1|ERG10
9	0.002875399	0.021040586	POL3|POL2|HYS2|APN2|DPB2|POL30|FPG1|POL32|OGG1
13	0.003064163	0.021040586	GNA1|GAL1|UAP1|PSA2|PMI1|CHT3|CHT2|GAL10|HEX1|DAC1|PMM1|SRB1|CHS1
9	0.003799425	0.024458797	TEL1|POL3|HYS2|RFA1|RAD54|RAD52|RAD51|POL32|RFA2
29	0.00412847	0.025013672	RPS13|RPS12|RPP1B|RPP1A|RPL6|RPS9B|RPL5|RPL9B|RPS3|RPS1|RPL28|YML6|RPS19A|RPS21|RPS42|RPL15A|RPL20B|RPS4A|RPL17B|RPS16A|RPP0|RPL10|RPL11|YST1|RPL8B|RLP24|RPS7A|RPL21A|RPS8A
16	0.005053554	0.028917558	ADE4|MET3|ADE6|RNR1|MET14|IMH3|PRS1|CDC19|PHO100|APT1|CYR1|GUK1|GUA1|AAH1|ADO1|RNR21
21	0.006061513	0.032859782	GPD1|CEK2|STE11|SST2|GSL2|SSK1|HST7|TEC1|YCK2|CLB2|HSL1|CDC42|FAR1|WSC2|STE4|GPD2|GRP2|GSL1|CPH1|SWI4|SSU81
11	0.008195076	0.042204639	SSB1|SOD3|HST3|SCH9|HSP104|HSP78|RIM15|TOR1|CYR1|TPK2|PNC1
13	0.00899723	0.044129271	MET6|MET15|MDH1-1|SER1|MEU1|SAH1|CHA1|CDG1|GCS1|SAM2|CYS4|CYS3|SER33
25	0.010403084	0.047949345	CAR1|DQD1|TDH3|SAM2|CYS3|CDC19|SHM1|GPM1|SER33|CYS4|ARG8|MET15|SER1|ILV5|IDH2|PFK2|LEU2|HIS3|LYS12|MET6|PRS1|ARG3|ARG4|GPM2|CHA1
8	0.010707135	0.047949345	LSC2|LSC1|ALD6|UGA1|ACS2|ERG10|GRP2|ACC1
8	0.0167196	0.071754951	PFK2|PSA2|PMI1|DAK2|SOU2|FBP1|PMM1|SRB1
10	0.01897164	0.075529589	SER1|GPM1|DAO1|SHM1|GPM2|CHA1|SER33|CYS4|HEM1|CYS3
6	0.019065721	0.075529589	IFE1|UGA2|UGA1|ERG13|ERG10|GAD1
4	0.022964282	0.087282263	CIS2|CDG1|GAD1|ECM38
6	0.024574618	0.087282263	FAS2|ACC1|FAS1|FAA4|ETR1|MRF1
6	0.024574618	0.087282263	FAD3|FAD2|OLE1|POT1-2|POT1|FEN1
9	0.030434453	0.103172624	XOG1|GSL2|MAL2|GSL1|TPS3|NTH1|GSY1|TPS2|GCA2
6	0.031051955	0.103172624	MET10|MET3|MET14|MET15|MET16|ECM17
11	0.03402549	0.109519546	LAT1|FDH3|CDC19|PFK2|ACS2|GPM2|GPM1|FBP1|GAL10|TDH3|PDC12
9	0.035552688	0.110967481	RNR21|URA7|PHO100|FCA1|URA3|CPA2|CDC21|DUT1|RNR1
8	0.048585947	0.147186841	NIT3|ADE4|GDH3|CPA2|ARG4|UGA2|UGA1|GAD1
5	0.0533843	0.154822985	PHO8|THI20|THI13|THI6|THI4
11	0.055407795	0.154822985	GPD1|SLC1|CDS1|GUT2|GPD2|PSD2|PLB5|PLB2|PLB1|DPP1|DPP3
6	0.056743547	0.154822985	ALD6|UGA1|POT1-2|POT1|ERG13|ERG10
4	0.058622295	0.154822985	MNT1|MNN2|MNT3|MNT2
4	0.058622295	0.154822985	MET3|MET6|TRR1|CYS3
9	0.08800992	0.226625544	LAT1|MDH1-1|CDC19|ACS2|ERG10|DLD1|GRP2|DLD2|ACC1
5	0.094416506	0.231545242	PFK2|MAL2|GAL1|GAL10|GCA2
5	0.094416506	0.231545242	ARG8|CAR1|ARG3|ARG4|GDH3
7	0.100391096	0.240471696	LAT1|MDH1-1|LSC1|SDH1|IDH2|SDH2|LSC2
3	0.13311508	0.298062027	CIS2|ECM38|SHM1
3	0.13311508	0.298062027	FEN1|ETR1|MRF1
3	0.13311508	0.298062027	DBF2|TEC1|YCK2
5	0.147452593	0.323140788	IDI1|SRT1|ERG13|ERG10|STE24
15	0.1513604	0.324794191	TIF4631|SEH1|PRT1|NMD3|MSN5|HCR1|RPG1A|GCD11|SUI1|GSP1|TIF35|TIF34|TIF|TIF3|TIF5
3	0.162603039	0.340067907	SNZ1|SNO1|SER1
14	0.165081508	0.340067907	VPS33|VAM3|VPS16|UME7|PCL5|SCH9|GCN2|RIM15|LCB2|PPH21|TOR1|TPK2|KCS1|ARP3
6	0.17190138	0.340496965	SLC1|GUT1|RHR2|DAK2|DPP1|DPP3
6	0.17190138	0.340496965	GOR1|MDH1-1|FDH1|ACS2|ERG10|SHM1
12	0.185124371	0.359770004	YPT31|SSB1|VPS21|VPS24|KIP4|WAL1|ENT3|SEC4|SEC7|VPS4|PEP7|CDC42
6	0.190480576	0.363324061	FCA1|PUT1|CAR2|CAR1|DAO1|AFP99
3	0.226295866	0.418630743	PHO8|ABZ1|PHHB
6	0.229739205	0.418630743	ALG8|ROT2|CWH8|ALG1|ALG6|ALG11
4	0.233130814	0.418630743	HEM1|HEM15|CYC3|MET1
7	0.243607305	0.418630743	VPS21|FAB1|SEC61|CUP5|SSH1|TUB2|TUB1
2	0.243862569	0.418630743	GPX3|GPX2
2	0.243862569	0.418630743	LIG4|CAS1
13	0.253498011	0.425577581	UTP4|SIK1|BMS1|IMP4|NOP1|NOP5|NOG1|NOP10|HRR25|GSP1|NHP2|NMD3|GAR1
3	0.259718677	0.425577581	HST6|CDR11|CDR4
4	0.260304734	0.425577581	IMP2|KAR2|SEC61|SSH1
13	0.266659254	0.429154737	YDJ1|KAR2|RAD23|SSB1|MSI3|ROT2|SEC61|IRE1|GCN2|CDC48|NPL4|SSH1|SAR1
5	0.279984836	0.443668278	FAA4|FDH3|POT1|POT1-2|ERG10
2	0.290419741	0.451529857	POT1-2|POT1
3	0.293713597	0.451529857	ILV5|CHA1|LEU2
4	0.316159338	0.478888408	HST3|PHO100|UGA2|PNC1
2	0.336587826	0.492806003	NCE103|GDH3
2	0.336587826	0.492806003	XKS1|ARD
4	0.34448575	0.492806003	PRS1|FBP1|SOL3|PFK2
4	0.34448575	0.492806003	FGR22|ALD6|PLC2|FAB1
5	0.353367034	0.498586364	HEX1|ALG11|MNN9|ALG1|MNN11
6	0.358556765	0.499072254	RPT6|RPN1|RPT4|RPN3|SCL1|PRE5
2	0.381803992	0.524344149	CEK2|CDC42
7	0.422754174	0.572943157	GRR1|UBC8|TOM1|CDC34|RAD6|CDH1|APC1
5	0.451869417	0.604448701	ILV5|IDH2|LEU2|ARG8|LYS12
3	0.525053783	0.692986741	CDS1|KCS1|FAB1
1	0.531514102	0.692986741	BIO2
3	0.554895847	0.714428403	ALD6|GAD1|UGA1
4	0.561990085	0.714629367	RPC40|RPO26|RPC19|RPC31
2	0.582330227	0.722650763	CDC21|SHM1
2	0.582330227	0.722650763	LCB2|ISC1
2	0.616246196	0.755635217	UGA2|ERG10
6	0.631588317	0.765336431	SOD3|PEX7|DAO1|POT1|POT1-2|FAA4
2	0.705210311	0.836581101	VAM3|SYN8
5	0.706626755	0.836581101	GLC7|PPH21|CDC55|SUP35|ERF1
2	0.730784804	0.84920876	FDH3|UGA2
4	0.733782327	0.84920876	UME7|SSK1|TOR1|WSC2
1	0.780673286	0.893437205	RIB5
2	0.796423907	0.893493117	PPH21|TOR1
7	0.802561873	0.893493117	SES1|KRS1|FRS1|MST1|HTS1|ALA1|THS1
1	0.806746212	0.893493117	LYS12
1	0.829722956	0.909164515	HIS3
5	0.845247127	0.916425833	HSP60|POP2|SKI3|SSC1|PFK2
1	0.88353646	0.9381882	ILV5
1	0.88353646	0.9381882	ARO10
1	0.920356886	0.957543022	ERG10
1	0.920356886	0.957543022	DQD1
1	0.93818721	0.966332826	MCD4
6	0.985114384	0.997807834	SDH1|SDH2|NUC2|NDH51|ALI1|CUP5
1	0.98812038	0.997807834	TBP1
2	0.999352108	0.999352108	DBP2|SSB1

Table S7 KEGG pathway analysis of upregulated differentially expressed genes associated with treatment with 7.8 µM Q-GRFT $^{lec\ neg}$ (3QG).

Gene name/ Description	Log2FC	P-value	Adjusted P-value
FRE7, Fre7p	-1.389	4.53E-44	2.82E-40
hypothetical protein CAALFM_C501070CA	-1.562	4.14E-36	1.29E-32
FRE30, Fre30p	-1.312	2.61E-33	5.42E-30
RNR1, ribonucleotide-diphosphate reductase subunit	-1.518	3.65E-32	5.67E-29
PGA45, Predicted GPI-anchored protein 45	-1.595	9.83E-32	1.22E-28
CTR1, high-affinity Cu transporter	-1.034	1.15E-29	1.19E-26
TRY6, Transcriptional regulator of yeast form adherence 6	-1.791	4.31E-29	3.76E-26
PHO84, phosphate transporter	-1.605	4.83E-29	3.76E-26
MET3, sulfate adenylyltransferase	-1.549	1.10E-27	7.59E-25
SOD3, Superoxide dismutase	-1.228	6.88E-23	4.28E-20
DNA-directed DNA polymerase alpha subunit	-1.138	4.81E-21	2.49E-18
CDC46, MCM DNA helicase complex subunit	-1.252	9.57E-21	4.58E-18
GDA1, guanosine diphosphatase	-1.099	1.58E-20	7.02E-18
putative ATPase	-0.908	2.06E-18	8.54E-16
GIT1, Glycerophosphoinositol permease 1	-1.922	4.06E-18	1.58E-15
GPX2, Glutathione peroxidase	-1.028	4.78E-18	1.75E-15
PRI2, DNA primase subunit	-1.385	2.21E-16	7.63E-14
SAM2, methionine adenosyltransferase	-0.887	2.97E-16	9.73E-14
DPB2, DNA polymerase epsilon noncatalytic subunit	-0.818	7.37E-16	2.23E-13
CDC54, MCM DNA helicase complex subunit	-0.946	7.91E-16	2.24E-13

Table S8. The top 20 down-regulated DEGs for 7.8 µM Q-GRFT $^{lec\ neg}$ treated *C. albicans* (3QG) vs non-treated control (VC) cells.

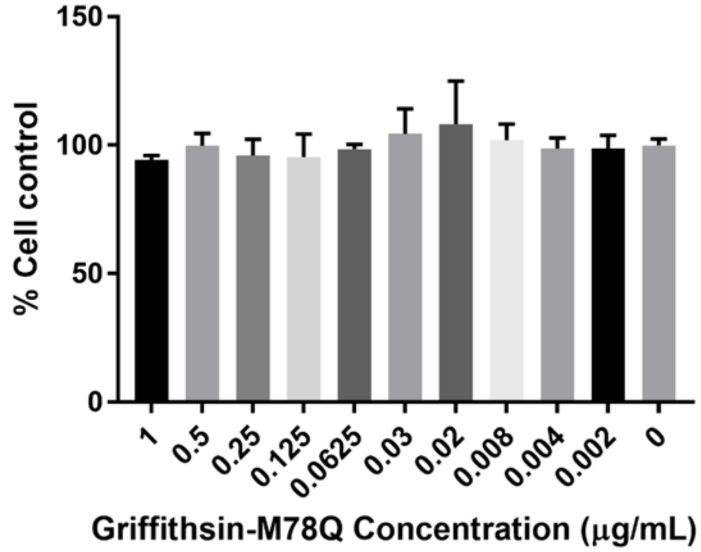

FIG S1 Q-GRFT cytotoxicity to McCoy cells used to culture *Chlamydia trachomatis*. Q-GRFT did not impede or inhibit the growth of McCoy cells.

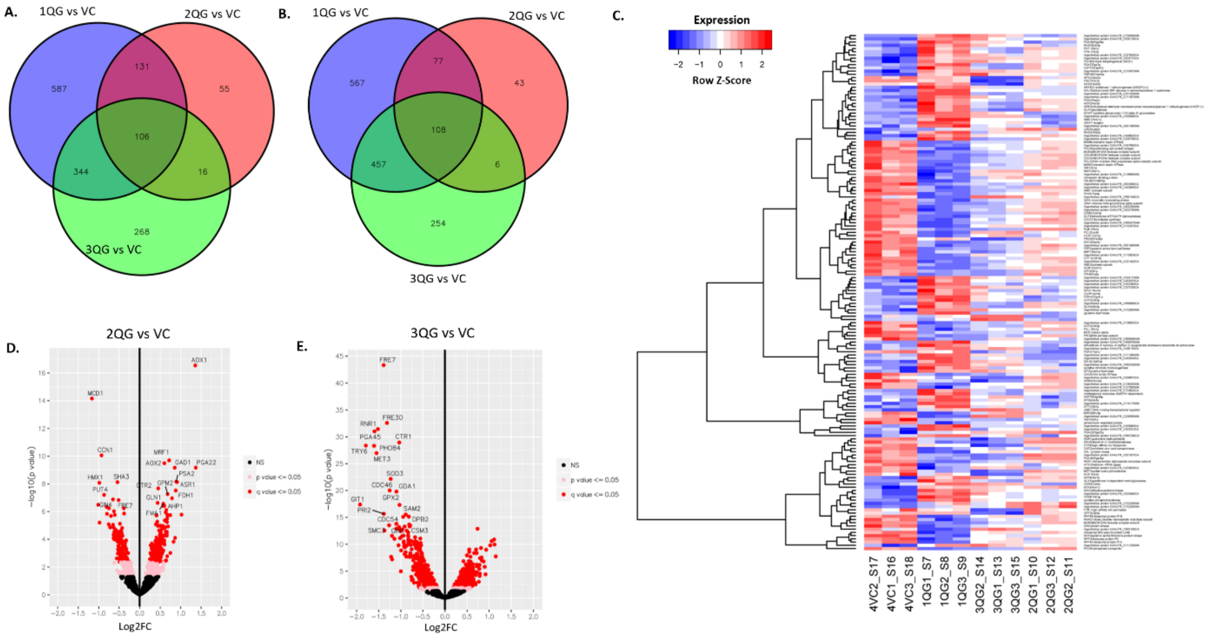

FIG S2 *C. albicans* RNA expression profile following treatment with Q-GRFT. *C. albicans* at a concentration of $1.0x10^5$ cells/mL was incubated overnight at 37°C in SDA medium with either 7.8 μM (1QG), 0.78 μM (2QG), 7.8 μM Q-GRFT $^{lec\ neg}$ (3QG) or control medium (VC). RNA was isolated and sequencing performed to determine expression under the different treatment conditions. Experiments were performed in triplicate. Venn diagrams showing shared differentially expressed genes for upregulated DEGs **(A)** and downregulated DEGs **(B)**. The intersections of the three respective circles represent overlapping DEGs among the three treatments. **(C)** Cluster analysis for genes showing a differential expression $|Log_2FC| \geq 2$ in the comparison demonstrating the z-score normalized expression (DESeq2 normalized counts). Volcano plots to examine the log2 fold change of DEGs at 2QG vs VC **(D)** and 3QG vs VC **(E)** treatments, for significance level p<0.05 and adjusted p value (q<0.05). Log2 fold change on the x-axis is plotted against -log10(p-value) on the y-axis.

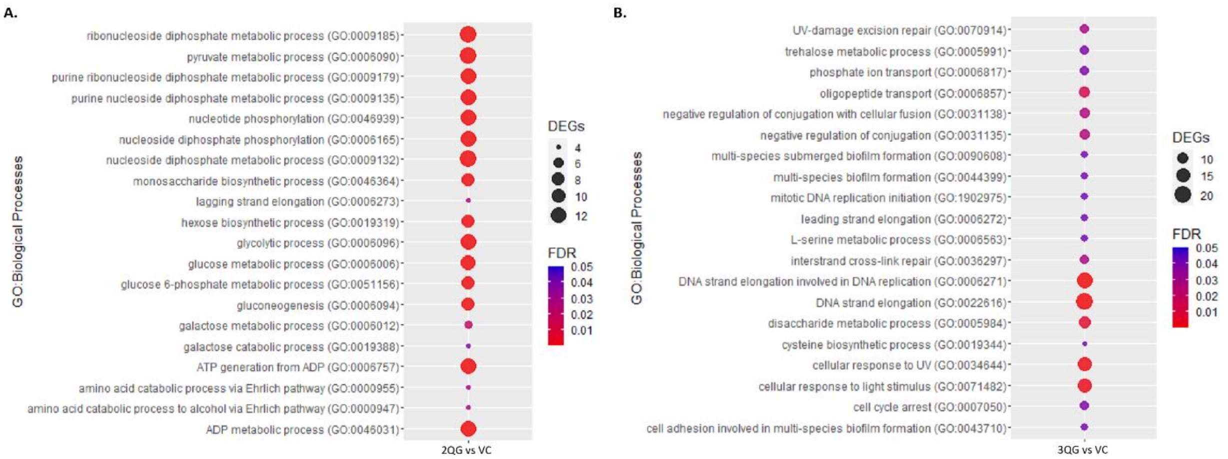

FIG S3 Top 20 enriched GO functions/ biological processes of DEGs with the adjusted p-values (FDR) for **(A)** 2QG vs VC and **(B)** 3QG vs VC treatment groups.
